# Screening of Bacterial Quorum Sensing Inhibitors in a *Vibrio fischeri* LuxR-Based Synthetic Fluorescent *E. coli* Biosensor

**DOI:** 10.3390/ph13090263

**Published:** 2020-09-22

**Authors:** Xiaofei Qin, Celina Vila-Sanjurjo, Ratna Singh, Bodo Philipp, Francisco M. Goycoolea

**Affiliations:** 1 Department of Bioengineering, Zhuhai Campus of Zunyi Medical University, Zhuhai 519041, China; iamxfqin@njtech.edu.cn; 2 Laboratory of Nanobiotechnology, Institute of Plant Biology and Biotechnology, University of Münster, Schlossplatz 8, D-48143 Münster, Germany; celina.vila@usc.es; 3 Department of Pharmacology, Pharmacy and Pharmaceutical Technology, University of Santiago de Compostela. Campus Vida, s/n, 15782 Santiago de Compostela, Spain; 4 Laboratory of Molecular Phytopathology and Renewable Resources, Institute of Plant Biology and Biotechnology, University of Münster, Schlossplatz 8, D-48143 Münster, Germany; singhr@uni-muenster.de; 5 Institute of Molecular Microbiology and Biotechnology, University of Münster, Corrensstraße 3, D-48149 Münster, Germany; bodo.philipp@uni-muenster.de; 6 School of Food Science and Nutrition, University of Leeds, Leeds LS2 9JT, UK

**Keywords:** compounds screening, quorum sensing inhibition, antibacterial, molecular docking

## Abstract

A library of 23 pure compounds of varying structural and chemical characteristics was screened for their quorum sensing (QS) inhibition activity using a synthetic fluorescent *Escherichia coli* biosensor that incorporates a modified version of lux regulon of *Vibrio fischeri*. Four such compounds exhibited QS inhibition activity without compromising bacterial growth, namely, phenazine carboxylic acid (PCA), 2-heptyl-3-hydroxy-4-quinolone (PQS), 1*H*-2-methyl-4-quinolone (MOQ) and genipin. When applied at 50 µM, these compounds reduced the QS response of the biosensor to 33.7% ± 2.6%, 43.1% ± 2.7%, 62.2% ± 6.3% and 43.3% ± 1.2%, respectively. A series of compounds only showed activity when tested at higher concentrations. This was the case of caffeine, which, when applied at 1 mM, reduced the QS to 47% ± 4.2%. In turn, capsaicin, caffeic acid phenethyl ester (CAPE), furanone and polygodial exhibited antibacterial activity when applied at 1mM, and reduced the bacterial growth by 12.8% ± 10.1%, 24.4% ± 7.0%, 91.4% ± 7.4% and 97.5% ± 3.8%, respectively. Similarly, we confirmed that *trans*-cinnamaldehyde and vanillin, when tested at 1 mM, reduced the QS response to 68.3% ± 4.9% and 27.1% ± 7.4%, respectively, though at the expense of concomitantly reducing cell growth by 18.6% ± 2.5% and 16% ± 2.2%, respectively. Two QS natural compounds of *Pseudomonas aeruginosa*, namely PQS and PCA, and the related, synthetic compounds MOQ, 1H-3-hydroxyl-4-quinolone (HOQ) and 1H-2-methyl-3-hydroxyl-4-quinolone (MHOQ) were used in molecular docking studies with the binding domain of the QS receptor TraR as a target. We offer here a general interpretation of structure-function relationships in this class of compounds that underpins their potential application as alternatives to antibiotics in controlling bacterial virulence.

## 1. Introduction

Bacteria communicate with secreted chemical signalling molecules that act as autoinducers in a phenomenon known as quorum sensing (QS) which allows them to fulfil a variety of functions, including bioluminescence, virulence and biofilm formation, among others [1]. In the case of Gram-negative bacteria, the autoinducer molecules belong mostly to the family of the acyl homoserine lactones (AHLs), which accumulate to particular threshold concentrations once the population of cells grow to sufficient [2,3].

AHL synthesis relies on the synthase LuxI family, while AHL reception depends on LuxR-type transcriptional regulators, which include the nominal LuxR protein from *Vibrio fischeri*, but also the related TraR and LasR from *Agrobacterium tumefaciens* and *P. aeruginosa*, among others. These act as transcriptional activators or repressors of the target QS genes [4,5,6,7]. The canonical LuxR protein from *V. fischeri* comprises two domains. The N-terminal domain is responsible of AHL binding and also can mediate protein dimerisation [8]. In contrast, the C-terminal domain contains a helix-turn-helix (HTH) motif, which is thought to make sequence-specific binding to DNA and to drive RNA polymerase binding to target promoters [9]. Much research has been conducted in recent years on mutant- LuxR-type proteins [10,11,12,13,14,15,16]. Among these, the TraR protein from *Agrobacterium tumefaciens* has been thoroughly studied, and its crystal structure has been solved, revealing the presence of the above mentioned two functional domains. In TraR, the N-terminal domain binds to N-3-oxooctanoyl-L-homoserine lactone (OOHL), and the C-terminal domain interacts with the DNA binding domain of the tra box [7,17]. TraR is a dimer in the presence of OOHL, and the TraR-OOHL-*tra* DNA ternary complex can be used as a prototype for the large family of AHL-induced transcription activators. The LasR protein of *Pseudomonas aeruginosa* shares 70% homology with TraR of *A. tumefaciens*, and the 3D model of the TraR active site closely resembles the X-ray structure of the LasR active site [18]. The signal binding sites in both apo-proteins are highly accessible, so TraR constitutes a useful model receptor which allows predicting the ability of putative QS inhibitors (QSIs) to block QS-based mechanisms in the human pathogen *P. aeruginosa* [19,20].

The development of strategies aimed to block or disrupt QS is gaining momentum. These efforts are directed to inhibit the production of virulence factors at the site of infection by dismantling the collective power of bacterial pathogens, an approach known as quorum quenching (QQ) or QS inhibition [21,22]. In the last years, QSIs have attracted significant attention from the scientific community and are considered as potential weapons and new generation antimicrobials in the therapeutic arsenal against infections caused by drug-resistant bacteria [23,24,25].

There are three major approaches to target bacterial QS using QSIs: (i) destruction of the signalling molecule, (ii) inhibition of signal production, and (iii) inhibition of the receptor [26]. To the date, most of the literature on QQ is centred in the investigation of AHL-degrading enzymes [27] and more abundantly, in QSIs that targeted to specific QS regulators of diverse species [26]. These QSIs may structurally resemble the natural ligand or may on the contrary, have an entirely different molecular structure [28,29]. QSIs include compounds of diverse sources, both natural and synthetic, and include fungal metabolites, plant substances, antibiotics, and synthetic derivatives of QS autoinducers or natural antagonists, to name a few [26,27,28,29]. Identification of QSIs is commonly performed through the screening of compound libraries and biosensor-based analysis of the QS response alone or combined with computer modelling analysis. These methods have allowed expanding the catalogue of available QSIs, which includes a variety of AHL analogues, brominated furanones, polyphenolic compounds and polypeptides [23,26,30,31,32].

Regarding computer modelling, docking-based screening of candidate QSIs is normally carried out by using a genetic algorithm on a library of 2344 compounds and calculates their binding free energy, hence identifying those candidates able to interact with target conserved residues in the binding site of a LuxR-type model receptor [33]. In-silico screening of ligand databases has thus become an important strategy towards the discovery of novel QSIs. GRID molecular interaction fields (GRID-MIFs) is accepted as an efficient method in virtual screening of candidate molecules which target protein binding sites. It is a computational procedure for detecting energetically favourable binding regions in proteins and small molecule drugs of known 3D structure. The energies are calculated using the electrostatic, hydrogen-bond, Lennard Jones, and entropic interactions of chemically selective probes with the chosen biological target. The program works by defining a three-dimensional grid of points that contains the chosen substrate binding site. The above mentioned calculations are repeated for each node in the three-dimensional grid and for each probe being considered. The results of these calculations are a collection of three-dimensional matrices, one for each probe-target interaction. A detailed description of the GRID program, the force field parameters, and details of calculations can be found elsewhere [34,35]. Briefly, a grid is projected inside the protein regions and cavities of interest. The probes are functional groups that can move stepwise from grid point to grid point. The calculated interaction energies of the probes are computed to create the MIFs, which represents the potential interaction of the protein with a particular chemical group. GRID-MIFs is considered as a high-throughput screening method to virtually analyse protein-ligand interactions [36,37].

In this study, we have screened 23 potential QS inhibition compounds, representing five groups according to their chemical structures. We found that seven of them were potent, dose-dependent inhibitors of the *V. fisheri* LuxR-based system expressed on recombinant *E. coli* biosensor cells, including two unprecedented ones. Another five compounds have shown antibacterial activity, while the remaining eleven compounds were inert at the tested doses. Moreover, we performed in silico GRID-MIFs-based computational molecular docking on the 3D crystal structure of TraR that allowed us to propose a hypothesis on the disparate effects observed experimentally for compounds of chemically related structure. We propose that our biosensor- and docking-based approaches have a high potential for drug designing purposes.

## 2. Results

### 2.1. Screening a Panel of Potential Quorum Sensing Inhibitors

**Figure 1:**
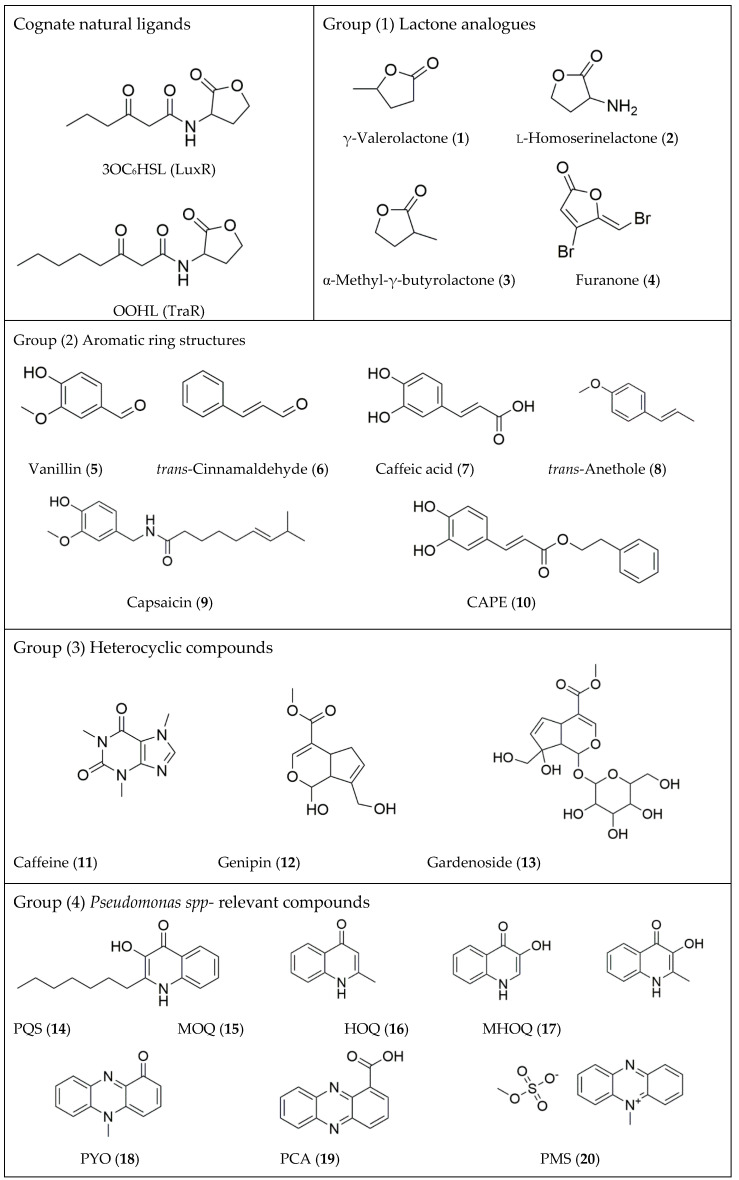

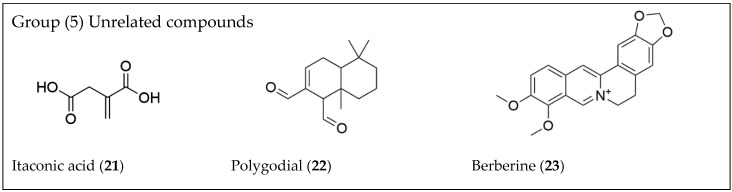
Chemical structures of studied compounds. Group (1) lactone analogues, Group (2) aromatic ring structures, Group (3) heterocyclic compounds, Group (4) *Pseudomonas* spp.-relevant compounds, and Group (5) structurally unrelated compounds. In the Figure are also shown the structure of natural LuxR and TraR ligands, namely 3OC6HSL and OOHL. Other details of the series of compounds are given in Materials and Methods section.

We selected a panel of 23 pure compounds with the potential to act as inhibitors of LuxR-AHL- mediated QS. These comprised the following groups, namely: Group (1) lactone analogues, Group (2) aromatic ring structures, Group (3) heterocyclic compounds, Group (4) *Pseudomonas spp-* relevant compounds, and Group (5) structurally unrelated compounds. Figure 1 shows the chemical structures of the cognate AHL molecules of LuxR and TraR regulators, namely *N*-3-oxohexanoyl-L-homoserine lactone (3OC_6_HSL) and OOHL, respectively.

All 23 compounds were tested using the *E. coli* Top10 pSB1A3-BBaT9002 biosensor, which expresses a synthetic version of the *lux* regulon of *V. fischeri* and produces a fluorescent as a response to external (3OC6HSL) [38]. The rate of the density-normalised fluorescence of the *E. coli* biosensor as a function of the 3OC6HSL concentration displays a Hill behaviour, with a *k*_Hill_ of 7.48 × 10^−10^ ± 9.03 × 10^−11^ M. At 3OC6HSL concentrations higher than 1 × 10^−9^ M, the fluorescence response is saturated, while the fluorescence levels are undetectable at a 3OC6HSL concentration of 1 × 10^−10^ M [38]. We evaluated the QS inhibitory effect of our panel of compounds by their ability to reduce the fluorescence response of this *E. coli* biosensor without compromising cell growth.

**Figure 2:**
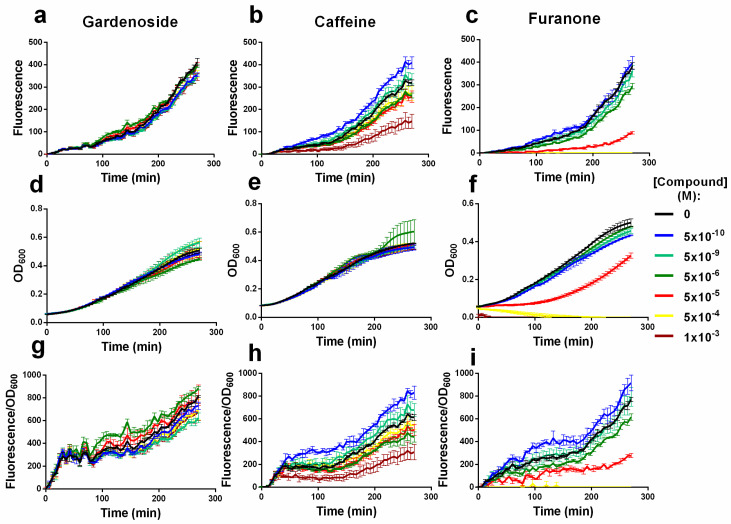
Effect of increasing concentrations of gardenoside, caffeine and furanone on the fluorescence (**a**–**c**), growth (**d**–**f**) and density-normalised fluorescence (**g**–**i**) of the *E. coli* biosensor over time. The three compounds are chosen as representatives of the following categories: no inhibition (gardenoside; **a**,**d**,**g**), QS inhibition in the absence of growth reduction (caffeine; **b**,**e**,**h**) and QS and growth inhibition (furanone; **c**,**f**,**i**). Results from additional experiments on other compounds are available in Supporting Information. Data shows the mean and standard deviation of a representative experiment with triplicated treatments.

Screening of the 23 compounds revealed that they could be classified into three main categories based on their QS inhibition activity, relative to their effects on bacterial growth. The first category refers to compounds that have not shown QS inhibition nor antibacterial activities (i.e., no apparent effect on the fluorescent response and growth of the *E. coli* biosensor). The second includes compounds with the ability to reduce the QS response of the biosensor without compromising cell growth. While the third one comprises compounds with the ability to reduce the QS response at the expense of hampering cell growth. Figure 2 shows representative dose-response curves of compounds belonging to the classes 1, 2, 3, namely gardenoside (13) (Figure 2a,d,g), caffeine (11) (Figure 2b,e,h) and furanone (4) (Figure 2c,f,i) on the fluorescence (Figure 2a–c), growth (Figure 2d–f) and density-normalized fluorescence (Figure 2g–i) of the *E. coli* biosensor. A selection of dose-response effects of other compounds is available as Supporting Information, as discussed below.

**Figure 3:**
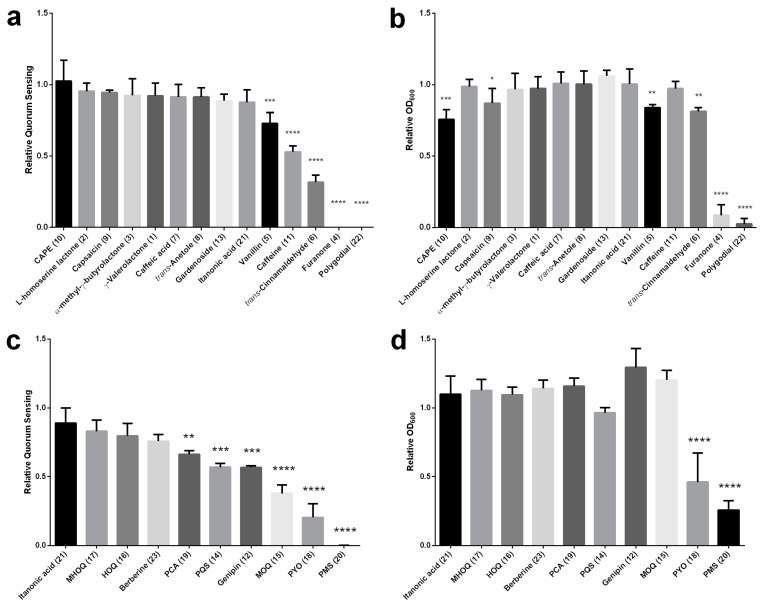
End-point effect of the 23 candidate compounds on the QS-based response and growth of the *E. coli* biosensor. (**a**) Effect of compounds **10, 2, 9, 3, 1, 7, 8, 13, 21, 5, 11, 6, 4, 22**; applied at 1 × 10^−3^ M on the density-normalised fluorescence of treated cells relative to control cells. Relative fluorescence was calculated as follows: the mean of the last ten values of density-normalised fluorescence over time, corresponding to 246–300 min of incubation (see Figure 2) was divided by the corresponding values of untreated cells. (**b**) Effect of compounds **10, 2, 9, 3, 1, 7, 8, 13, 21, 5, 11, 6, 4, 22;** applied at 1 × 10^−3^ M on cell density of treated cells relative to control cells. Relative OD_600_ was calculated as follows: the mean of the last 10 OD_600_ values over time, corresponding to 246–300 min of incubation (see Figure 2) was divided by the corresponding values of untreated cells. (**c**)**.** Effect of compounds **21, 17, 16, 23, 19, 14, 12, 15, 18, 20;** applied at 5 × 10^−5^ M on the density-normalised fluorescence of treated cells relative to control cells. Relative fluorescence was calculated as in (**a**,**d**) Effect of compounds **21, 17, 16, 23, 19, 14, 12, 15, 18, 20**; applied at 5 × 10^−5^ M on cell density of treated cells relative to control cells. Relative OD_600_ was calculated as in (**b**) *t*-Student statistical comparisons were made using itaconic acid as a reference treatment (* *p* < 0.05, ** *p* < 0.01, *** *p* < 0.001 and **** *p* < 0.0001). Data show the mean and standard deviation of three independent experiments with triplicated treatments.

Next, we calculated the end-point effect of the 23 candidate compounds on the QS-based response and growth of the biosensor, relative to those of untreated cells. Figure 3 shows end-point, relative reduction of density-normalised fluorescence and cell density of compounds tested at fixed concentrations of 5 × 10^−^^5^ M (Figure 3a,b) and 1 × 10^−3^ M (Figure 3c,d). The rationale behind the chosen concentrations was based on the maximum water solubility of the compounds. Relative values close to 1.0 indicate none or negligible effect of a given compound on the density-normalised response and/or growth of the biosensor. In turn, we considered that relative fluorescence values significantly lower than 1.0 as were a diagnose of inhibition of the QS-based response, proven that the relative OD_600_ values stayed close to 1.0. This condition applied to vanillin (**5**), caffeine (**11**) and *trans*-cinnamaldehyde (**6**) applied at 1 × 10^−3^ M (Figure 3a,b); and to phenazine carboxylic acid (PCA, **19**), 2-heptyl-3-hydroxy-4-quinolone (PQS, **14**), genipin (**12**) and 1*H*-2-methyl-4-quinolone (MOQ, **15**) applied at 5 × 10^−5^ M. In all cases, the relative QS and OD_600_ data were compared statistically against itaconic acid, due to its negligible effect on both parameters.

Figure 3a shows that the compounds *trans*-cinnamaldehyde (**6**) (*p* < 0.0001), caffeine (**10**) (*p* < 0.0001), and, to a less extent, vanillin (**5**) (*p* < 0.001) can significantly reduce the QS-based fluorescence of the biosensor when applied at 1 × 10^−3^ M. Importantly, the QS inhibitory effect of caffeine (10) was not accompanied by any compromise of cell growth (Figure 3b). As for vanillin (**5**) (*p* < 0.01) and *trans*-cinnamaldehyde (**6**) (*p* < 0.01), they slightly reduced cell growth by 16.0% and 18.6%, respectively (Figure 3b). Furanone (**4**) (*p* < 0.0001), and polygodial (21) (*p* < 0.0001), which abolished the QS-based fluorescence of the biosensor at the tested concentration, they concomitantly exerted a dramatic antibacterial effect (cf. Figure 3a,b). Figure 3c shows that compounds PCA (**19**) (*p* < 0.01), PQS (**14**) (*p* < 0.001), genipin (**12**) (*p* < 0.001) and MOQ (**15**) (*p* < 0.0001) significantly reduced the density-normalized fluorescence of the biosensor when tested at 5 × 10^−^^5^ M (Figure 3a) and, importantly, they did not exert a significant effect on bacterial growth (Figure 3b). By contrast, compounds PYO (**18**) (*p* < 0.0001) and PMS (**20**) (*p* < 0.0001), showed a strong deleterious effect both on the QS response of the biosensor and its growth (cf. Figure 3c,d). We carried out a series of experiments to confirm that inhibition of the density-normalised fluorescence observed with PYO (**18**) and genipin (**12**) at sub-lethal doses was in fact due to interference with the QS response of the biosensor and not due to GFP fluorescence quenching. To this end, we used a control *E. coli* strain Top10 pBCA9445-jtk28282::sfGFP, which constitutively expresses a super folded version of GFP (sfGFP). We compared the effects of both compounds in the *E. coli* biosensor and control strains (Figures S2–S8). In our hands, neither of them at concentrations in the range 1 × 10^−^^8^ M to 1 × 10^−^^4^ M (Figures S3–S5) acted as quenchers of sfGFP fluorescence in the control strain *E. coli* Top10 pBCA9445-jtk28282::sfGFP. We observed a high degree of experimental variability and not apparent dose-response effects of PYO at sub-lethal concentrations ranging from 1 × 10^−8^ to 1 × 10^−4^ M (Figure S5). The implications of these disparate observations are further considered in the Discussion section.

### 2.2. Computation of GRID-MIFs

**Figure 4:**
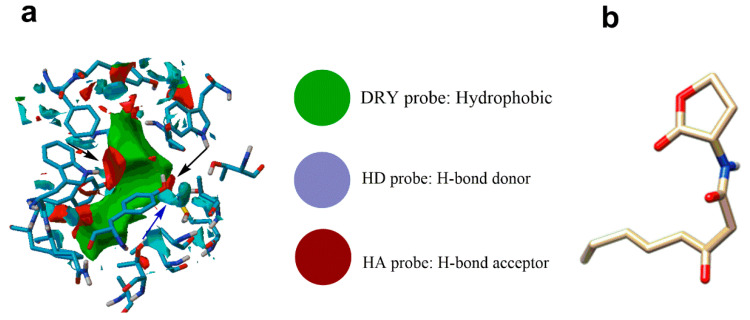
(**a**) GRID-MIFs for TraR protein with DRY probe (green) showing favorable hydrophobic interaction sites. GRID-MIFs for TraR protein with HD (blue) and HA (red) probes showing favorable hydrogen bond (blue for hydrogen bond donor and red for hydrogen bond acceptor) binding sites. (**b**) Natural substrate OOHL.

The three-dimensional molecular crystal structure of TraR receptor was obtained from the protein data bank (PDB No. 1L3L). Information about the binding region where the natural ligand OOHL interacts with TraR was described elsewhere [17]. Favorable interaction points at the binding site of the receptor was studied with GRID-MIFs. To define the GRID maps at TraR binding site, the Autogrid utility inbuilt in AutoDockTools 1.5.6 software was applied. Three different chemical probes (HD, HA, DRY) were used, representing the potentially significant functional group at the binding site. In Figure 4a the GRID-MIFs generated at TarR binding site is shown. Here, the green patch generated by DRY probe accounts for the favourable hydrophobic interaction ligand and receptor; blue contours were generated by HD (donor) probe responsible for favourable hydrogen bonds between receptor and ligand, and red contours were generated by HA (acceptor) probe, which informs about favourable hydrogen bonds between ligand and receptor amino acid residues. Comparing with the natural substrate OOHL (Figure 4b) the dry probe matches with the ring and all carbons in the substrate, the HD (blue) probe matches the NH group of substrate and HA (red) matches the O functional group present in the substrate.

### 2.3. Docking and Interaction of Selected Compounds

**Figure 5:**
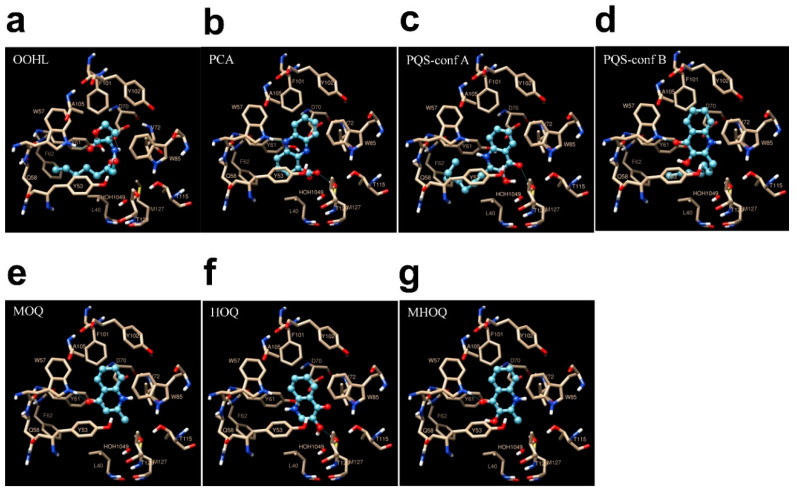
Interaction of TraR receptor with (**a**) OOHL; (**b**) PCA, (**c**) and (**d**) PQS; (**e**) MOQ; (**f**) HOQ and (**g**) MHOQ. (**c**) shows us the PQS conf A and (**d**) shows PQS conf B. Close-up view of all compounds binding site, oxygen carbon and nitrogen are colored in red yellow and blue, respectively, hydrogen bonds are shown as blue line.

We docked six selected compounds, based on their generated GRID-MIFs, over the binding domain of TraR. We included the cognate OOHL ligand as a reference. This enabled to gain Information on dock score, hydrogen bond and hydrophobic interactions. We selected ligand conformations using that of the natural ligand OOHL into the binding site as a reference (Figure 5a). Thus, we fixed docked ligand binding poses to that of the natural ligand. In other words, superimposition of the docked ligand on OOHL conformation in TraR’s crystal structure implied that the aromatic ring of the ligand coincided with the lactone ring of the natural substrate. Table 1 lists the estimated binding energy values for the docked compounds. Strikingly, PCA (19) and PQS (14) showed a more negative docking score (i.e., predicted free energy of binding) than that estimated for the cognate ligand OOHL, which would in principle indicate a stronger affinity of these two compounds towards the binding site of TraR (Table 1). Figure 5b shows the docking-predicted binding pose of PCA into TraR’s binding pocket. Two ligand conformations were identified for PQS (14), namely PQS-conf A (Figure 5c) and PQS-conf B (Figure 5d and Table 1). Both conformations were similar to OOHL binding pose, only differing in ring flip, which affects hydrogen bond interactions between ligand and receptor (cf. Figure 5a–c). The estimated docking score for PQS (14)-conf A was more negative than that of OOHL, whereas the predicted value for PQS-conf B was less negative, indicating a potential stronger affinity of conf B vs conf A towards the binding site of TraR (Table 1). Docking scores of smaller ligands, namely MOQ, HOQ and MHOQ were less negative than that of OOHL, predicting poorer affinities for these ligands towards TraR (Table 1).

We studied in detail the docking-predicted poses of selected candidates to elucidate the mechanisms of receptor-ligand possible interactions (Table 1). Figure 5 shows interaction maps of the six selected compounds (Table 1) with TraR’s binding pocket. Clear from Figure 5a are the presence of four hydrogen bonds established between the cognate substrate OOHL and specific amino acids at the binding pocket of the receptors The H- bonds are located as follows: (1) between Trp 57 in TraR and keto group present in the lactone ring, (2) between Tyr 53 in TraR and the 1-keto group of the lactone, (3) between Asp 70 in TraR and the imino group in OOHL and (4) between Tyr 61 in TraR and the 3-keto group of OOHL (Figure 5a). Apart from these bonds, the cognate ligand also establishes strong hydrophobic interactions with nearby residues, as shown in Table 1. Altogether, it seems that both hydrophobic and hydrogen bond interactions are important for ligand selectivity. Our docking results confirm previous docking predictions of binding and pose conformations for OOHL in TraR’s binding site [18]. Figure 5b shows the docking-predicted map for PCA (19)-TraR interaction (19). Importantly, our predictions show the presence of three hydrogen bonds with TraR’s binding pocket, namely: (1) between Trp 57 in TraR and the deprotonated N present in the aromatic group of PCA, (2) and (3) between Thr 129 in TraR, an intermediate water molecule and the carboxylic acid functional group of PCA (Figure 5b). Again, our docking predicted several hydrophobic interactions between specific aminoacids of the binding site of TraR and the aromatic and heterocyclic rings of PCA (19) (Table 1). Figure 5c shows the predicted maps for PQS-confA and the binding site of TraR. In this case, two hydrogen bonds are predicted to form, namely: (1) between Thr129 and the keto group present in the aromatic ring of PQS (14), (2) between a hydroxyl group in PQS and a molecule of water (Figure 5c). In turn the predicted map for PQS-conf B and TraR interaction (Figure 5d shows one hydrogen bond formed between Trp 57 in TraR and the keto group present in the aromatic ring ofPQS (14). Both PQS-conf A and -confB are expected to establish strong hydrophobic interactions with the binding pocket of TraR, as listed in Table 1 and the long carbon chain of PQS in both poses cover most of the hydrophobic patch generated by GRID (cf. Figure 4 and Figure 5c,d). Panels e, f and g of Figure 5, illustrate the prediction interaction maps for TraR and MOQ (15), HOQ (16) and MHOQ (17), respectively. Docking-predicted poses in the binding site of the receptor are very similar (cf. Figure 5e–g); hence, the predicted interaction sites for the three of them did not very much. However, in the case of both MOQ (15) and MOQH (17) an hydrogen bond was predicted between Trp 57 in TraR and the keto group in the aromatic ring of the ligands (cf. Figure 5e,g), while this interaction was not observed in HOQ (15) (Figure 5f). Strikingly, a relative inhibition of the QS-based response of the *E. coli* biosensor was found to be significantly higher for MOQ (15) than that of HOQ (16) and MOQH (17) (Figure S9). This, in principle would indicate that the specific position of H-bonding between these kind of molecules and the binding site of TraR would not necessarily explain *per se* towards LuxR-based proteins.

## 3. Discussion

QSIs are gaining momentum as potent alternatives to the use of classical antibiotics in the context of rising resistance spread and with enormous potential in many fields, from food science to agriculture and medicine [39,40]. QSIs are structurally diverse molecules that can be synthesised or extracted from natural sources. Here, we screened a total of 23 chemically diverse compounds with potential QS inhibition activity to identify their impact on LuxR-regulated QS models. To this end, we used a well-established *E. coli* Top10 biosensor assay and in silico modelling to recreate the structural interactions between the ligand candidates and the LuxR-like receptor.

The candidate compounds were classified into five broad groups according to with their chemical structure (Table 1). Group 1 comprised the alkyl-substituted lactones (Table 1): γ-valerolactone (**1**), L-homoserine lactone (**2**), α-methyl-γ-butyrolactone (**3**), and furanone **4**. In this group, only furanone (**4**) showed a significant inhibition (≥37.5%) of the fluorescent- QS-based response of the biosensor at concentrations ≥ 5 × 10^−6^ M 37.5% (Figure 3a and Figure S10). At 1 × 10^−3^ M, furanone completely abolished the QS-based response of the biosensor, albeit a dramatic, deleterious effect on cell growth (Figure 3b and Figure S10).

γ-Butyrolactones were the first class of small signalling molecules identified from Gram-positive species *Streptomyces griseus*. They were found to induce streptomycin production and sporulation [41]. Even though the chemical structure of γ-butyrolactones is rather similar to that of AHLs, except for the carbon side-chain, it is known that the receptors of γ-butyrolactone do not bind to AHL receptors and that AHL do not bind to γ-butyrolactone receptors. Also, both receptors have low structural similarity [41]. The functions of the signalling molecules also differ as AHLs i.a. are known to regulate QS in Gram-negative bacteria, while γ-butyrolactones mainly regulate the production of antibiotics and differentiation.

From our results, we found that neither α-methyl-γ-butyrolactone, γ-valerolactone nor L-homo-serine lactone showed any QS inhibition activity. These results are consistent with the notion that the carbon side chain is important for binding to the LuxR receptor. Also, it is not unexpected that γ-valerolactone did not show activity, as the methyl substituent is in a different position of the lactone ring than that in 3-oxohexanoylhomoserine. As suggested in previous studies, our results support the proposal that the LuxR receptor does not recognise α-methyl-γ-butyrolactone nor other related lactones (e.g., γ-valerolactone).

Halogenated furanones which resemble AHLs structurally were first discovered in the marine red alga *Delisea pulchra* and proved to preventing swarming motility with the opportunistic human pathogen *Proteus mirabilis* and *S. liquefaciens* without affect growth rate [42,43]. These compounds were the first QSIs found to occur in nature. After that, Manefield et al. further obtained proof-of-principle of the QS inhibition activity of the same type of halogenated furanones by specifically interfering with AHL-mediated gene expression at the level of the LuxR protein [44]. Furthermore, Ren et al. found that (5*Z*)-4-bromo-5-(bromomethylene)-3-butyl-2(5*H*)-furanone is a non-specific intercellular signal antagonist [45]. Later, Defoirdt et al. proved that this furanone disrupts quorum sensing-regulated gene expression in *Vibrio harveyi* by decreasing the DNA-binding activity of the transcriptional regulator protein LuxR [46]. Besides halogenated furanones with side alkyl chains that have QS inhibition activity, some synthetic halogenated furanones lacking alkyl chain also have anti-biofilm formation activity through interference with QS, and have been found to protect surfaces from being colonised by *S. epidermidis* [47]. Compound (5*Z*)-4-bromo-5-(bromomethylene)-3-butyl-2(5*H*)-furanone included in our library, a synthetic derivate of natural furanone has been reported as a potent antagonist of bacterial QS that not only can increase bacterial susceptibility to tobramycin and SDS when it is applied for action with *P. aeruginosa* biofilms but also can inhibit virulence factor expression because of targeting QS systems [48]. Several previous studies have shown that halogenated furanones can interact with the LuxR protein and induce conformational changes due to rapid proteolytic degradation of the furanone-LuxR complex, which in turn destabilises the AHL-dependent transcriptional activator [49]. However, independent studies confirmed that halogenated furanones without alkyl chains were strongly toxic to the planktonic cell. While furanones with long alkyl chains were shown not to reduce the biofilm formation [50,51]. The present study confirmed that furanone without alkyl chains has high toxicity against the *E. coli* Top10 pSB1A3-BBaT9002 strain, as it reduced 50% the bacterial growth when dosed at 5 × 10^−^^5^ M. In fact, at 1 × 10^−^^3^ M, indeed, furanone can kill all bacteria, diagnostic that the lack of alkyl chain is at play in the induced toxicity. It has been suggested that the increase of water solubility may explain this effect [51]. Interestingly, when dosed at 5 × 10^−^^6^ M, the results showed that furanone could reduce the fluorescence production at no detrimental expense of the bacterial growth (Figure 2c). A possible explanation to these phenomena might be that some compounds when applied at sub-lethal concentrations will not kill bacterial but delay the QS activity because bacteria become weaker than at the normal condition. This phenomenon gave us the illusion that those compounds were QSIs, but when we increased the dose, we noticed that they had an antibacterial activity. The observed antimicrobial effect at furanone concentrations ≥ 5 × 10^−6^ M (Figure S10) prompted us to search QSIs with lower associated toxicity. Closer attention should be paid to this phenomenon to discriminate between potential QSIs more carefully.

Group 2 candidates included compounds with at least one aromatic ring. Vanillin (**5**), proposed as a less toxic alternative to brominated furanones, revealed a relative reduction of the biosensor’s fluorescent response of 27.1 % when applied at 1 × 10^−3^ M (Figure 3a and Figure S7), an effect that was accompanied by a discrete, 16.0% reduction of cell growth (Figure 3b and Figure S7). Belonging to the same group, the well-reported QSI, *trans*-cinnamaldehyde (**6**) revealed a potent capacity to reduce the QS-based response of the biosensor to up to 68.3% (Figure 3a and Figure S7) when applied at 1 × 10^−3^ M, with a slight reduction of cell growth (18.6%; Figure 3b and Figure S7). Surprisingly, related compounds caffeic acid (**7**) and *trans*-anethole (**8**) were apparently innocuous to the *E. coli* biosensor even at concentrations of 1 × 10^−3^ M (Figure 3a,b) while capsaicin (**9**) and CAPE (**10**) inhibited cell growth by 12.8 and 24.4%, respectively (Figure 3b).

Vanillin (**5**) is a well-known food flavouring agent and is a major constituent of vanilla pods. Its QS inhibition activity has recently been demonstrated in *Aeromonas hydrophila*, *Agrobacterium tumifaciens* NTL-4, *Chromobacterium violaceum* CV026 when applied at 250 µg/mL (1.64 mM), where it showed significant inhibition in short-chain AHLs (C4-HSL (69%) and 3OC8-HSL (59.8%)), followed by C6-HSL(32%), and C8-HSL (28%), but lower inhibition in long-chain AHLs (C14-HSL (13.5%) and C10-HSL (12%)). It also reduces the biofilm formation on the reverse osmosis membrane of *A hydrophila* [52]. Our own experiments confirmed that vanillin at 1 × 10^−^^3^ M can inhibit the QS activity with 3OC6HSL 27.1%. It has been speculated that the possible mechanism whereby vanillin inhibits QS activity is that it interferes with the binding of the short-chain AHLs to their cognate receptor [52]. Our study offers experimental evidence that vanillin may also interfere with binding of 3OC6HSL to its cognate LuxR receptor.

*trans*-Cinnamaldehyde (**6**) is a component of cinnamon and cassia essential oils, and it is commonly present in food as a flavouring agent and fungicide. In a previous study, it was shown that 200 µM cinnamaldehyde can reduce by 70% the fluorescence intensity due to the expression of GFP, induced by 3-oxo-C6-HSL in a bioreporter *E. coli* ATCC 33,456 pJBA89, and also effective at inhibiting AI-2 mediated QS [53]. Furthermore, Brackman et al. proved *trans*-cinnamaldehyde at concentrations < 1 mM shifts the SDS-PAGE mobility of LuxR-DNA. They concluded that *trans*-cinnamaldehyde and cinnamaldehyde derivatives interfere with AI-2 based QS in various *Vibrio* spp. by decreasing the DNA-binding ability of LuxR [54]. Recent molecular docking analysis studies suggested that *trans*-cinnamaldehyde interacts with LasI and EsaI substrate binding sites thus inducing the QS inhibition activity [55]. Our own studies also confirm that *trans*-cinnamaldehyde inhibits the LuxR-mediated GFP production in the *E. coli* Top 10 biosensor by 68.3% at 1 × 10^−^^3^ M concentration. This strain does not express the LuxI gene, thus it cannot produce LuxI synthase of 3-oxo-C6-HSLs but can constitutively overexpress LuxR protein. In our related study, we have shown that *trans*-cinnamaldehyde not only inhibits the expression of GFP, but it also retards its kinetics [38]. We suggest that there are maybe two mechanisms at play for QS inhibition involving LuxR. Firstly, the three-carbon aliphatic side chain of *trans*-cinnamaldehyde could interfere with the binding of 3OC6HSL to LuxR receptor; secondly, it can also decrease the DNA-binding ability of LuxR dimers, as previously suggested.

*trans*-Cinnamaldehyde (**6**)-related compounds, namely caffeic acid (**7**) and *trans*-anethole (**8**), were also tested on their QS inhibition activity. The results showed that neither of them had QS inhibition activity nor antibacterial effect at the tested concentration of 1 × 10^−^^3^ M. Caffeic acid is a phenolic acid that has various documented beneficial biological properties. Besides being a powerful antioxidant, it also has anticancer, anti-inflammatory and antiviral activities [56]. *trans*-Anethole, in turn, is also a natural component of anise seeds and fruits and it is used as a flavouring ingredient. Until now, its antibacterial and QS inhibitory activities have not been tested.

Comparing the chemical structures of caffeic acid and *trans*-anethole with that of *trans*-cinnamaldehyde, we can clearly see that neither the catechol phenolic nor the aromatic ester groups of caffeic acid and trans-anethole, respectively, have any effect on increasing the binding affinity with the LuxR protein. These results may reflect the importance of the unsubstituted aromatic ring in *trans*-cinnamaldehyde to make π-π interactions with the receptor residues at the binding site of LuxR. In this regard, the phenolic residues of caffeic acid can act as the H-bond donors, but not acceptors, at the hydrogen bond acceptor binding domain of the binding pocket of LuxR, hence, decreasing its overall binding efficiency. Similarly, in trans-anethole, the presence of the methyl ester substituent at the aromatic ring may be is enough to prevent its efficient binding with the binding site of LuxR. However, vanillin, with one phenolic, one methyl ester and an aldehyde substituent at the aromatic ring, does show QS inhibition activity as discussed above. Therefore, our study is consistent with the idea that the role of the presence of H-bond acceptor groups along with the the π-π interactions of the aromatic ring, is what determines the overall affinity of compounds to bind with LuxR receptor.

Capsaicin (**9**), structurally related to vanillin (**5**), is a natural alkaloid extracted from fruit of Capsicum family and it is responsible for the pungency of chili peppers. Its structure has an aromatic ring and a long hydrophobic aliphatic chain with a polar amide group. Most studies with capsaicin have addressed its pharmacology and clinical applications [57]. Only a few studies have focused on the inhibition activity of the growth of the gastric pathogen *Helicobacter pylori* [58,59]. One such study found that capsaicin did not inhibit the growth of a human fecal commensal *E. coli* strain [58]. So far, the QS inhibitory activity of capsaicin has not been documented. Our results show that even at the concentration of 1 × 10^−^^3^ M, capsaicin did not significantly reduce the QS activity of the *E. coli* Top10 pSB1A3-BBaT9002 biosensor, and it decreased the bacterial growth only by 12.8% (*p* < 0.05). If we compare the structures of capsaicin with that of vanillin, we can observe that they share identical substitution positions in their aromatic ring, except that vanillin has not the long aliphatic chain, but only a strong aldehyde H-bond acceptor group. Our results indicate that the increase in the length of the aliphatic chain does not lead to an increase in binding affinity. CAPE (**10**) is the main active component of propolis extract. Its chemical structure is described as the ester of caffeic acid and phenetyl alcohol; hence, it is a catechol ring with an ester chain bearing another aromatic ring. CAPE has known bioactivities such as antimicrobial, anti-inflammatory and cytotoxic activities. About the antimicrobial activity of CAPE, it has been reported that can inhibit the 60% *E. coli* growth when the concentration is over 60.6 µM. This has been explained as the result of the synthesis of reactive oxygen species that damage the outer membrane of the bacteria-induced by CAPE [60,61]. Our experiments contrast with this study, as we found that CAPE only inhibits the growth of the *E. coli* Top10 pSB1A3-BBaT9002 strain by 24.4% when applied at 1 × 10^−^^3^ M. As a possible explanation for the observed discrepancies between the results of our study and the previous ones may stem in the distinct protocols of the assays to quantify the bacterial growth rate. Overall, no QS inhibition activity was shown on the surviving bacteria after treatment with CAPE.

Group 3, comprised by heterocyclic compounds, revealed candidates with important QSI capacity, namely caffeine (**11**) and genipin (**12**), which have been shown to reduce the QS-based response of the biosensor by 47% and by 43.3% when applied at 1x10^−3^ M and 5x10^−5^ M, respectively (Figure 3a,c, Figures S7 and S8) and with negligible effects on cell growth (Figure 3b–d, Figures S7 and S8). Belonging to the same group, gardenoside (**13**) did not show any apparent effect on the *E. coli* biosensor at the doses tested (Figure 3a,b).

Caffeine (**11**) is yet another alkaloid which occurs in coffee cherry and tea. Documented uses of caffeine include as a pesticide to kill certain larvae, insects and beetles. Some studies have shown that caffeine, applied at 2.5 mg/mL (≈12.8 mM), can retard the growth of *E. coli*, *Enterobacter aerogenes, Proteus vulgaris*, and *P. aeruginosa* within a short time. The first time caffeine was tested as QS inhibition compound against *C. violaceum* CV026 and *P. aeruginosa* PA01 strains, it was found that when applied at 0.3–1.0 mg/mL (≈1.5–5.0 mM), it inhibits violacein production in *C violaceum* CV026, and short chain AHLs production and swarming motility in *P. aeruginosa* PA01 [62]. Our experiments also proved that caffeine, applied at 1 × 10^−^^3^ M to the *E. coli* Top10 pSB1A3-BBaT9002 biosensor strain, it can inhibit 47% GFP production without affecting bacterial growth. Our results, along with previous studies [62], suggest that caffeine has a broad spectrum of QS inhibition activities in different bacterial species; interestingly, all share in common to contain the AHL-regulated QS system. As earlier reported by Norizan et al. Caffeine did not degrade C6-HSL [62], so we hypothesise here that it can be a competitor that binds with AHL receptors because the keto groups from the aromatic ring can also form strong hydrogen bonds with type I QS LuxI/LuxR receptors.

Also in Group 3, genipin (**12**) is an iridoid compound isolated from *Gardenia jasminodies* Ellis fruits. It is the aglycone derivative of geniposide. It was initially identified as a protein cross-linking agent but can also inhibit the production of nitric oxide by downregulating the activity of nuclear factor-κB (NF-κB) [63]. It is also a cell-permeable inhibitor with anti-inflammatory and anti-angiogenic activity mediated by the induction of apoptosis in hepatoma and hepatocarcinoma cell lines [64]. A number of studies have also shown that genipin cross-links chitosan nano- and microparticles, thus allowing them to be used for QS inhibition and the delivery of antimicrobial drugs [65,66]. We found, for the first time, that genipin on its own can inhibit GFP production in the *E. coli* Top10 pSB1A3-BBaT9002 biosensor, without exhibiting significant toxicity, more effectively than a diverse spectrum of alternative compounds. Knowing that genipin possesses inherent fluorogenic properties, we wondered whether the observed effect on the fluorescent response of our biosensor could be due to an artefact associated with the direct fluorescence quenching [67]. We performed a series of extra experiments where we tested genipin on the control *E. coli* strain Top10 pBCA9445-jtk28282::sfGFP strain (Supporting Information). We found that genipin concentrations ranging from 9.95 × 10^−10^ to 1.19 × 10^−4^ M did not exert significant fluorescence quenching on the control strain (Figures S2 and S3). Nevertheless, the fact that the fluorescence over growth profiles of both bacterial strains widely differ (Figure S1) make these comparisons a difficult task. Moreover, both strains express different GFP species (namely GFPmut3b and sfGFP in the biosensor and control strain, respectively) and their expression vectors widely diverge (see Supporting Information) [68,69]. Future efforts should be focused on validating the effects of genipin by using a control *E. coli* Top10 strain expressing GFPmut3b constitutively from a modified version of the pSB1A3-BbaT9002 plasmid. On our hands, genipin and related compounds are promising candidates for the development of novel therapeutic approaches based on the inhibition of QS in bacteria. Our data showed a significant difference between the QS inhibition activities of genipin and the closely related glycosylated iridoid compound, gardenoside. These two iridoids share similar structures, differing only with respect to the glycosylation in the hydroxyl group of C1, and in the hydroxyl group at position C8 of the heterocyclic structure, in gardenoside. This result may have a biological significance in host-pathogen interactions via inhibition of QS, as glucose oxidase (GOD) is synthesised by many plants, fungi and bacteria. This hypothesis would need further experimental validation.

Quinolone- and phenazine- like compounds belonging to Group 4 (Table 1) are reported to play important roles in QS-regulated phenotypes of *Pseudomonas spp* [70]. These comprise the alkylquinolone PQS (**14**) signal of *P. aeruginosa* and three structurally-related synthetic quinolones, namely MOQ (**15**), HOQ (**16**) and MHOQ (**17**) (Table 1); bearing a bicyclic core structure with different substituents. Among these three structurally-related synthetic compounds, only MOQ demonstrated significant relative QS inhibition activity of 62.2%. The rest of tested compounds, namely HOQ and MHOQ, did not have any detectable activity, neither QS inhibition nor antibacterial (Figure 3c,d and Figure S9). Also, compound PQS comprised not only one six-carbon ring but also a long carbon chain at the six-member heterocyclic ring, which showed an important QSI activity, by inhibiting the QS-based response of the biosensor by 43.1%, with a negligible effect on cell growth (Figure 3c,d and Figure S8). Compounds PYO (**18**), PCA (**19**) and PMS (**20**) are similarly with three rings, but only PYO and PMS have high GFP inhibition and high toxicity, compound PCA also has QQ activity, inhibiting GFP production by 33.7%, but no toxicity (Figure 3c,d).

Group 4, comprised heterocyclic compounds, some of which can be produced naturally, such as the *Pseudomonas* spp. metabolites (PQS, PCA, PYO), while the rest are synthetic [70]. It is well known that phenazines have antimicrobial activity [71]. Moreover, some of these compounds are known to act as signalling molecules that regulate the QS systems in *P. aeruginosa* as discussed in detail below.

Compound PQS has been found as a QS signal that participates in the *P. aeruginosa* QS network and acts as a link between las and rhl quorum sensing, which either directly or indirectly, mediates the expression of 182 genes in *P. aeruginosa* [72,73]. Besides the intraspecific signalling role of PQS in *P. aeruginosa* involving interactions with its cognate LasR-like PqsR receptor and non-cognate-LuxR-like-RhlI, there is no evidence to the date on PQS interference with other LuxR-based QS regulation circuitries. Despite the fact that there is currently no crystal structure available for RhlI, it is known that it shows significant sequence divergence from TraR, our prototype for docking studies. Thus, it is unknown how PQS binds to RhlI in *P. aeruginosa*. A recent paper from Mukherjee et al. explored ligand-receptor binding of PqsR with C4HSL, by generating a homology model based on the *E. coli* SdiA structure, which is a LuxR-like closest homolog of PqsR [74]. In SdiA and other LuxR-type proteins, the highly conserved amino acids Trp68 and Asp81 interact with the amide group-oxygen and the amide group-nitrogen, respectively, of the cognate C4HSL. Other conserved residues, such as Tyr72 and Trp96, are required for hydrophobic and van der Waals interactions with the ligands [74]. These interactions seem to correlate with our docking predictions of OOHL establishing H-bonding with Trp57 and hydrophobic interactions with Tyr53 and Trp85 in the binding site of TraR (Figure 5a and Table 1). A similar scheme of PQS predicted H-bonding and hydrophobic interactions with Trp57 and Tyr61 (Figure 5c,d and Table 1) would in principle serve as a rationale for a strong affinity of PQS to LuxR homologs and the observed quorum quenching effect on the biosensor’s LuxR-regulated response (Figure 3c and Figure S8).

Phenazines are a well-known family of pigments that are secreted from *P. aeruginosa*. Among phenazines, PYO is widely known due to its cytotoxic and redox activities [75]. Importantly, PYO is a terminal signalling factor in the QS network of *P. aeruginosa* [76]. PYO is also an intercellular signal that triggers specific responses in enteric *E. coli* and *Salmonella enterica*, via the SoxR regulon. Whether this kind of signal transduction is also involved in our *E. coli* biosensor in the presence of PYO is a question that we cannot elucidate at present [77]. Also, it is well known that PYO interacts with molecular oxygen to form ROS species that change the redox balance of the cells and that GFP fluorescence can be affected by the presence of ROS species [78,79]. To shed some light on extra effects of PYO on fluorescence quenching and/or the metabolism of *E. coli* Top 10 cells, we decided to perform extra experiments applying increasing PYO concentrations on both the *E. coli* Top10 pSB1A3-T9002 (Figure S4) biosensor and the *luxR*-deficient *E. coli* Top10 pBCA9445-jtk28282::sfGFP (Figures S5 and S6). Strikingly, we found a lack of dose-response effect of PYO on the fluorescence of both strains (Figures S4–S6), together with strong variability among experiments (cf. Figures S5 and S6). These disparate results could arise from some of the transductory and/or redox activity of PYO on our *E. coli* Top10 cells and need further investigation.

PCA (**19**) is yet another redox-active phenazine pigment that is produced from *P. aeruginosa* [80]. PCA is known to be a broad-spectrum activity compound that inhibits the growth of several plant pathogenic species (e.g., *Corynebacterium fascians, Agrobacterium tumefaciens, Erwinia aroideae, Diplodia zeae*) [81,82]. Further studies have found that PCA is precursor for more complex phenazine metabolites, such as 1-hydroxyphenazine, phenazine-1-carboxamide and PYO [76]. Yun Wang et al. found that PCA may shift the redox equilibrium between Fe(III) and Fe(II) in *P. aeruginosa* [83]. Even though PCA and PYO, both are redox-active phenazines, in our study, we found that, at 5 × 10^−5^ M, PYO has strong toxicity to *E. coli* Top 10 pSB1A3-BBaT9002 strain, while PCA only inhibits the fluorescence intensity but has no effect on bacterial growth. The different effect of PYO and PCA can be attributed to the fact that PCA may help the *E. coli* alleviate Fe(III) limitation by reducing it to ferrous iron [Fe(II)], thus promoting the bacterial growth [83], thus allowing to observe the QS inhibition activity. We will discuss further this aspect in the context of the in silico molecular docking results below.

PMS is a simple phenazonium salt and an electron acceptor and carrier in biochemical oxidation/reduction studies [84,85]. It is also a O_2_^−^ generating agent that can increase intracellular H_2_O_2_ levels and lead to the formation of free radicals that can affect bacterial growth [85]. This explains why in our experiments PMS shows strong toxic effect against *E. coli* Top 10 pSB1A3-BBaT9002 strain. As explained above, this strain is lacking the SoxR regulon to confer resistance against redox stress.

Previous studies have proved that phenazines are antibiotic compounds that can inhibit microbial growth because of the redox-active effect. From our own studies, it can be argued that when phenazines (namely, PCA, PYO, PMS) are applied at the same concentration of 5 × 10^−5^ M, only PYO and PMS are toxic to the *E. coli* Top10 pSB1A3-BBaT9002 strain, as we mentioned above. Interestingly, PCA, does not inhibit bacterial growth but has QS inhibition activity. Several studies have also noticed this phenomenon. Morales et al. found that lower concentrations of PCA, PYO and PMS inhibited the fungal yeast-to-filament transition and affected the development of *C. albicans* wrinkled colony biofilms but allowed growth. However, those phenazines have anti-candida activity when the concentration is higher than 500 µM, which means that those compounds have different biological effects at different concentrations [86]. Furthermore, Skindersoe et al. used sub-inhibitory concentrations of antibiotics in *P. aeruginosa* and found that lower doses of antibiotics could modulate gene expression, so that they interfere with QS signalling [87]. To the best of our knowledge, this dual concentration-dependent activity of phenazines had not been reported before to operate in a mutant *E. coli* Top10 pSB1A3-BBaT9002 strain. However, recently, it has been hypothesised that it is a general mechanism of action of many compounds [88].

For the identification of functional group and their arrangement in the binding site required for binding ligand, GRID map was generated by using three different chemical probes i.e., H bond donor (HD), H bond acceptor (HA) and DRY probe. Grid-MIFs generated for TraR indicated (Figure 4) that acceptor interaction points and hydrophobic patches are dominant in comparison to donor interactions points at the binding. Comparing the functional group present in ligands with the GRID-MIFs (Figure 4) it is clear that because of big hydrophobic patch in the center of cavity, hydrophobic interaction from carbon either from aromatic ring or long chain carbon make very favorable interaction. Apart this one donor group at one side of aromatic ring makes favorable interaction with the Trp 57. Hydrogen bond interaction with Trp 57 is identified as important interaction in receptor substrate interaction. Apart from HOQ we found this interaction in all other five ligands. Whereas at the other side of ring one donor or one acceptor would also make favorable interaction with the Thr129 or water.

Consequently, we identified that in the same ringside two functional groups e.g. an OH group just next to the O (acceptor group), do not make a favourable interacting group. This can be observed in the docking score results for HOQ and MHOQ both with lower scores than MOQ. This might explain that in vitro HOQ and MHOQ, did not exhibit QS inhibition activity, while MOQ, that lacks the 3-hydroxyl group did exhibit QS inhibition activity experimentally. PQS showed better score, i.e., PQS-conf A (−8.04) and PQS-conf B (−6.59), in comparison to HOQ and MHOQ because of the long alkyl chain and an overall more favourable hydrophobic interaction (Table 1). In the PQS-conf A (−8.04), the H-bond between the Trp 57 and ligand is missing in this conformation, but because of PQS has a longer alkyl chain than OOHL, a H-bond with Thr 129 and putative H-bond with water, PQS-conf A has a better score than the other compounds. Whereas in PQS-conf B (−6.59) the H-bond is present between ligand O and Trp 57, but because of the OH just next to O is not a favourable interaction (according to GRID-MIFs), hence its lower score. This analysis clearly indicates that OH next to O is not a favourable functional group for interaction in ligand PQS, which affects the pose and docking score. Moreover, because of the H-bond interactions and more hydrophobic interactions in comparison to natural ligand OOHL, this compound is better over other ligands. Along with PQS, PCA has also shown a high dock score compared to other ligands. We argue that this is the result of the combined effect of the three aromatic rings making more hydrophobic interaction, the deprotonated N present in the aromatic group involved in H-bond interaction with Trp57, and the carboxylate function group involved in H-bond interaction with Thr129 and also probably with water. These favourable interactions might explain the high QS inhibition activity observed for PQS and PCA.

Finally, Group 5 contained a more diverse collection of organic molecules with complex structures. Experimentally, with the exception of compounds itaconic acid at 1 × 10^−3^ M and berberine at 5 × 10^−5^ M, none of these compounds showed QS inhibition activity. However, we found that polygodial exhibited strong bacterial growth inhibition. Polygodial is a bicyclic sesquiterpene dialdehyde, isolated from different traditional medicinal plants such as *Polygonum hydropiper* and *P. punctatum* [89]. Kubo et al. showed that polygodial has the antibacterial activity against various bacteria, not only as a surfactant to form the pyrrole with primary amine groups at the plasma membrane, thereby disturbing the balance of the membranes, but also may react with various intracellular components when it enters into the cells after the membrane damaged [89]. We also proved that polygodial has high antibacterial activity that suppressed almost completely the growth of *E. coli* Top10 pSB1A3-BBaT9002 strain when dosed at 1 × 10^−3^ M.

Berberine is an isoquinoline-type alkaloid isolated from *Coptidis rhizomaand* (“huang lian” in Chinese), a plant used in traditional Chinese medicine, and from other plants. It has been reported that when the concentration is at 30-45 µg/mL could exhibit an antibacterial effect and inhibit biofilm formation of *Staphylococcus epidermidis*. Whether the biofilm formation inhibition of berberine observed in Gram-positive bacteria is connected with the QS regulation is not confirmed [90]. However, recent studies have shown that berberine inhibits the QS in Gram-negative bacteria including antimicrobial-resistant *E. coli* strains, *P. aeruginosa PA01*, *C. violaceum* and *Salmonella enterica* [91,92]. Sun et al. investigated the QS inhibition activity of berberine in antimicrobial-resistant (AMR) *E. coli* strains and found that berberine inhibited biofilm formation and downregulated QS-related genes *luxS*, *pfS, hflX*, *ftsQ*, and *ftsE* of AMR *E. coli* strains at 1/2 (640 µg/mL) or 1/4 (320 µg/mL) minimal inhibitory concentration (MIC) [90]. Thus the tested berberine concentrations of berberine were tested by Sun et al. were ≥ 9.5 times higher than ours (cf. ≥ 160 µg/mL and 16.8 µg/mL in Sun et al.’s and our study, respectively). Moreover, the AMR *E. coli* QS system is a LuxS/AI-mediated system, unrelated to the LuxR-based circuitry present in our biosensor. Under our setting, we found no QS inhibition at a berberine concentration of 50µM (16.8 µg/mL). The lower concentration tested in our assays may explain the observed lack of QS inhibition activity. We decided to limit berberine concentration to 50 µM due to solubility problems at higher concentrations. Further efforts should be focused on testing the QS inhibition potential of berberine and other related compounds at concentrations comparable to those of Sun et al.’s and exploring whether the strong effect observed on LuxS-based QS systems can be extrapolated to LuxR-regulated circuitries.

## 4. Materials and Methods

### 4.1. Library of Tested Chemical Compounds

Compounds were selected according to their chemical structure and were divided into five groups. They were in all cases of high purity (≥90%) and were either commercially available or synthesised. They were shipped in glass vials as powders or in liquid form and were dissolved in water or organic solution (ethanol or methanol) before use. The details for each compound are given in Table 2. Each is assigned a reference number used throughout this manuscript. 3-Oxohexanoyl-homoserine lactone (3OC6HSL) and all other chemicals were of analytical grade and, unless otherwise stated, were purchased from Merck KGaA (Darmstadt, Germany).

### 4.2. Bacterial Strains

The QQ activity of the 23 selected compounds was determined using the *E. coli* Top 10 strains listed below. The BioBrick standard biological sequence BBa_T9002, ligated into vector psb1a3 (http://partsregistry.org/Part:BBa_T9002), was a gift from Prof. Anderson (UC Berkeley, CA, USA). The sequence BBa_T9002 was introduced by chemical transformation into *E. coli* Top 10 (Invitrogen, Life Technologies Co., Leicestershire, UK) and single-colony cultures from the transformed strain were stored as 30% glycerol stocks at −80 °C as described in Section 2.3 below. The sequence BBa_T9002 comprised the transcription factor (LuxR), which is constitutively expressed, but it is active only in the presence of the exogenous autoinducer signalling molecule 3OC6HSL. At an adequate concentration, two molecules of 3OC6HSL bind to two molecules of LuxR and activate the expression of GFP (output), under the control of the lux pR promoter from *Vibrio fischeri*. The fluorescence biosensor was calibrated for different 3OC6HSL concentrations, as described in our previous studies [4]. An *E. coli* strain Top10 (Invitrogen, Life Technologies Co., U.K.) was transformed with plasmid pBCA9445-jtk2828, carrying a superfolder version of the *gfp* gene (*sfgfp*), which was kindly donated by Prof. Anderson Lab (UC Berkeley, Berkeley, CA, USA). The transformed strain expresses sfGFP constitutively and was used as control culture to test possible fluorescence quenching artefacts of genipin and PYO that could account for the effects observed in the fluorescence *E. coli* Top10 pSB1A3-BBaT9002 biosensor (Supporting Information).

### 4.3. Growth Media and Glycerol Stocks Preparation

Bacterial strains were cultivated using on Luria-Bertani (LB) and M9 minimal medium purchased from BD GmbH (Heidelberg, Germany). We inoculated 10 mL of LB broth supplemented with 200 µg/mL ampicillin with a single colony from a freshly streaked plate of Top10 containing BBa_T9002 and incubated the culture for 18 h at 37 °C, shaking at 100 rpm. Glycerol stocks were prepared as described in our previous studies [38]. Briefly, a 500 µL aliquots of overnight bacterial culture were mixed with 500 µL 30% sterile glycerol in 1.5 mL plastic vials and stored at −80 °C. Prior to each experiment, an aliquot of a glycerol stock from the single culture was diluted 1:1000 into 20 mL M9 minimal medium supplemented with 0.2% casamino acids, 1 mM thiamine hydrochloride and 200 µg/mL ampicillin (AppliChem GmbH, city, Germany). The culture was maintained under the same conditions until the OD600 reached ~0.15 (~5 h).

### 4.4. E. coli Top10 Fluorescent Biosensor Assay

Each tested compound was dissolved in MilliQ water or 100% organic solution (ethanol or methanol) according to their solubility at a high concentration of 200 mM, then diluted with MilliQ water to produce samples at six concentrations: 2 × 10^−^^2^, 1 × 10^−^^2^, 1 × 10^−^^3^, 1 × 10^−^^4^, 1 × 10^−^^7^ and 1 × 10^−^^8^ M; however, some compounds can only be prepared at a maximal concentration of 50 µM given by their water solubility. The 3OC6HSL was dissolved in acetonitrile to a stock concentration of 100 mM and stored at –20 °C kept in a sealed glass vial. Prior to each experiment, serial dilutions from the AHL stock solution were prepared in water to produce solutions with a concentration ranging from 100 mM to 10 nM. We then mixed 10 µL 3OC6HSL solution with 10 µL of the diluted compounds at different concentrations in the wells of a flat-bottomed 96-well plate (cat. # M3061, Greiner Bio-One, city, state abbrev if USA, country) and each well was then filled with 180 µL aliquots of the bacterial culture to test for QS inhibition activity. The final inhibitor concentrations, therefore, ranged from 1 × 10^−^^3^ M to 5 × 10^−^^10^ M. Several controls were also set up. Blank 1 contained 180 µL of M9 medium and 20 µL of MilliQ water to measure the absorbance background. Blank 2 wells contained 180 µL of bacterial culture and 20 µL of MilliQ water to measure the absorbance background-corrected for the cells. Finally, positive control wells contained 10 µL of water plus 10 µL 3OC6HSL solution and 180 µL of the bacterial culture to measure the fluorescence background. The plates were incubated in a Safire Tecan-F129013 Microplate Reader (Tecan, Crailsheim, Germany) at 37 °C and fluorescence measurements were taken automatically using a repeating procedure (λ_ex_ = 480 nm and λ_em_ = 510 nm, 40 µs, 10 flashes, gain 100, top fluorescence), absorbance measurements (OD_600_) (λ = 600 nm absorbance filter, 10 flashes) and shaking (5 s, orbital shaking, high speed). The interval between measurements was 6 min. For each experiment, the fluorescence intensity (FI) and OD_600_ data were corrected by subtracting the values of absorbance and fluorescence backgrounds and expressed as the average for each treatment. Data were presented as FI/OD_600_ versus incubation time. All measurements were taken in triplicate.

### 4.5. Protein Structure File, Ligand Database

The X-ray crystal structure of *Agrobacterium tumefaciens* TraR was downloaded from the Protein Data Bank (PDB ID 1L3L) and used for computer docking. All the water molecules were removed except one molecule in the binding pocket, which plays an important role in interaction and forms the hydrogen bond with the autoinducer OOHL of TraR protein. To define the grid box of TraR protein, OOHL was used as a ligand to select spheres and also followed with the Information from the previous study [18]. The 2D structures of six compounds (OOHL, MOQ, HOQ, MHOQ, PCA and PQS) were drawn manually using Marvin sketch v6.1.3 (ChemAxon Ltd., Budapest, Hungary) and saved as MDL mol files. The mol files were merged into a single mol file and likewise converted to 3D structures using Discovery Studio 3.5 client software. PyMol was used for visualisation and molecular modelling.

### 4.6. Molecular Docking Studies

For the generation of GRID-MIFs (molecular interaction fields) at the TarR binding site where a given chemical group can interact favourably, Autogrid program inbuilt in AutoDockTools 1.5.6. was used. For MIF generation, mainly three probed were applied i.e., DRY probe representing hydrophobic interaction, HA probe to representing H bond acceptor groups, and HD probe to representing H bond donor groups. Docking guided by the grid map was performed using Autodock tool. Fifty conformations were generated for each docked substrate. Binding scores between the ligand and protein was evaluated using the autodock utility autoscorer considering the hydrogen bond forces, electrostatic forces, van der Waals forces, solvation energy and entropy.

### 4.7. Statistical Analysis

All the experiments were performed in triplicates to validate reproducibility and the P values were calculated statistically by Student’s *t*-test. Values were expressed as mean ±SD. A comparison analysis was performed between tests and control.

## 5. Conclusions

In this study, we have screened the QS inhibition activity of a library of 23 structurally different compounds against an *E. coli* Top10 pSB1A3-BBaT9002 reporter of AHL-regulated QS. This library included a selection of natural and synthetic compounds that occur naturally in plants and in bacteria species such as *P. aeruginosa*. We were able to establish cues of structure-function relationships for compounds with QS inhibitory activity (e.g., *trans*-cinnamaldehyde, vanillin, caffeine, PQS, PCA). We showed, for the first time, that genipin and MOQ have QS inhibition activity. We also conducted molecular simulations using GRID-MIFs on a selection of compounds (e.g., MOQ, HOQ, MHOQ). Our results aid in the future rational design of novel QS inhibition compounds. For example, the introduction of a 3-methyl group in MOQ may increase the binding affinity substantially to the TraR receptor and hence the QS inhibition activity. This hypothesis could be validated experimentally in future studies. The results of this study may pave the way to future works aimed to fully realise the potential of QS inhibition as an alternative strategy to overcome antimicrobial resistance and biofilms in clinical and other settings.

## Figures and Tables

**Table 1:** Cumulative results for the compounds docked against TraR protein.

Compound	Hydrogen Bonding Interactions	Dock Score (Kcal/mol)	Hydrophobic Interactions
OOHL	4 (including Trp 57)	−7.58	Leu 40, Tyr 53, Gln 58, Trp 85, Phe101, Ala 105, Ile 110.
MOQ (**15**)	1 (including Trp 57)	−6.16	Tyr 53, Val 72, Trp 85, Phe 101, Tyr 102, Ala 105, Met 127
HOQ (**16**)	0	−5.72	Trp 57, Tyr 61, Asp 70, Val 72, Trp 85, Phe 101, Met 127
MHOQ (**17**)	1 (including Trp 57)	−5.83	Tyr 53, Asp 70, Val 72, Trp 85, Phe 101, Tyr 102, Ala 105, Met 127, Thr 129
PCA (**19**)	3 (including Trp 57)	−8.01	Leu 40, Tyr 53, Tyr 61, Val 72, Trp 85, Phe101, Tyr 102, Ala 105, Met 127
PQS (**14**) (conf A)	2	−8.04	Leu 40, Tyr 53, Tyr 61, Val 72, Val 73, Trp 85, Phe 101, Tyr 102, Ala 105, Met127, Thr 129
PQS (**14**) (conf B)	1 (including Trp 57)	−6.59	Ala 38, Leu 40, Ala 49, Thr 51, Gln 58, Tyr 61, Phe 62, Val 72, Trp 85, Phe 101, Met127

**Table 2:** List of screened compounds.

Number	Compounds	Group ^1^	Solvent/Method	Supplier
1	γ-Valerolactone	Group (1) Lactone analogues	Water	Sigma (St. Louis, MO, USA)
2	L-Homoserine lactone		Water	Santa Cruz Biotechnology
3	α-Methyl-γ-butyrolactone		Water	Sigma (St. Louis, MO, USA)
4	Furanone((*Z*-)-4-Bromo-5- (bromomethy-lene)-2(5*H*)-furanone)		First dissolved in ethanol, then diluted with water	Sigma (St. Louis, MO, USA)
5	Vanillin	Group (2) Aromatic ring structures	Water	Sigma (St. Louis, MO, USA)
6	*trans*-Cinnamaldehyde		First dissolved in ethanol, then diluted with water	Sigma (St. Louis, MO, USA)
7	Caffeic acid		First dissolved with ethanol, then diluted with water	Sigma (St. Louis, MO, USA)
8	*trans*-Anethole		Water	Sigma (St. Louis, MO, USA)
9	Capsaicin		First dissolved with ethanol, then diluted with water	Merck KGaA (Darmstadt, Germany)
10	CAPE (caffeic acid phenethyl ester)		First dissolved with ethanol, then diluted with water	Merck KGaA (Darmstadt, Germany)
11	Caffeine	Group (3) Heterocyclic compounds	water	Merck KGaA (Darmstadt, Germany)
12	Genipin		Water	Challenge Bioproducts Co., Ltd.
13	Gardenoside		water	Nanjing Zelang Medical Technology Co.,Ltd
14	PQS (2-heptyl-3-hydroxy-4-quinolone)	Group (4) Quinolone- and phenazine-based compounds relevant to QS systems of *Pseudomonas* spp	First dissolved with methanol, then diluted with water	Merck KGaA (Darmstadt, Germany)
15	MOQ (1*H*-2-methyl-4-quinolone)		First dissolved with methanol, then diluted with water	Prof. Fetzner’s ^2^
16	HOQ (1*H*-3-hydroxyl-4-quinolone)		First dissolved with methanol, then diluted with water	Prof. Fetzner’s ^3^
17	MHOQ (1*H*-2-methyl-3-hydroxyl-4-quinolone)		First dissolved with methanol, then diluted with water	Prof. Fetzner’s ^4^
18	PYO (pyocyanine)		First dissolved with methanol, then diluted with water	Merck KGaA (Darmstadt, Germany)
19	PCA (Phenazine carboxylic acid)		First dissolved with methanol, then diluted with water	Key Organics Ltd. (Camelford, UK)
20	PMS (Phenazine methosulfate)		First dissolved with methanol, then diluted with water	Sigma (St. Louis, MO, USA)
21	Itaconic acid	Group (5) Structurally unrelated compounds	First dissolved in ethanol, then diluted with water	Sigma (St. Louis, MO, USA)
22	Polygodial		Water	Santa Cruz Biotechnology
23	Berberine		First dissolved in ethanol, then diluted with water	Sigma (St. Louis, MO, USA)

^1^ The Group column refers to the classification based on chemical structural features (Figure 1), as explained in the text; ^2^ Synthesised by Prof. Susane Fetzner according to the method of Eiden et al. [93]. HPLC and UV absorption analysis indicated a purity of over 90%; ^3^ Synthesised by Prof. Susane Fetzner according to the method of Evans and Eastwood [94]. HPLC and UV absorption analysis indicated a purity of over 90%; ^4^ Synthesised by Prof. Susane Fetzner according to the method of Cornforth and James [95]. HPLC and UV absorption analysis indicated a purity of over 90%.

## Supplementary Materials

The following are available online at https://www.mdpi.com/1424-8247/13/9/263/s1.

## References

[1] Fuqua W.C., Winans S.C., Greenberg E.P. (1994). Quorum Sensing in Bacteria: The LuxR-LuxI Family of Cell Density-Responsive Transcriptional Regulatorst. J. Bacteriol..

[2] Rutherford S.T., Bassler B. (2012). Bacterial Quorum Sensing: Its Role in Virulence and Possibilities for Its Control. Cold Spring Harb. Perspect. Med..

[3] Bassler B., Losick R. (2006). Bacterially Speaking. Cell.

[4] Passador L., Cook J., Gambello M., Rust L., Iglewski B. (1993). Expression of Pseudomonas aeruginosa virulence genes requires cell-to-cell communication. Science.

[5] Ochsner U.A., Reiser J. (1995). Autoinducer-mediated regulation of rhamnolipid biosurfactant synthesis in Pseudomonas aeruginosa. Proc. Natl. Acad. Sci. USA.

[6] Singh P.K., Schaefer A.L., Parsek M.R., Moninger T.O., Welsh M.J., Greenberg E.P. (2000). Quorum-sensing signals indicate that cystic fibrosis lungs are infected with bacterial biofilms. Nature.

[7] Vannini A., Volpari C., Gargioli C., Muraglia E., Cortese R., De Francesco R., Neddermann P., Di Marco S. (2002). The crystal structure of the quorum sensing protein TraR bound to its autoinducer and target DNA. EMBO J..

[8] Hanzelka B.L., Greenberg E.P. (1995). Evidence that the N-terminal region of the Vibrio fischeri LuxR protein constitutes an autoinducer-binding domain. J. Bacteriol..

[9] Stevens A.M., Dolan K.M., Greenberg E.P. (1994). Synergistic binding of the Vibrio fischeri LuxR transcriptional activator domain and RNA polymerase to the lux promoter region. Proc. Natl. Acad. Sci. USA.

[10] Chai Y., Winans S.C. (2004). Site-directed mutagenesis of a LuxR-type quorum-sensing transcription factor: Alteration of autoinducer specificity. Mol. Microbiol..

[11] Choi S.H., Greenberg E.P. (1991). The C-terminal region of the Vibrio fischeri LuxR protein contains an inducer-independent lux gene activating domain. Proc. Natl. Acad. Sci. USA.

[12] Kiratisin P., Tucker K.D., Passador L. (2002). LasR, a Transcriptional Activator of Pseudomonas aeruginosa Virulence Genes, Functions as a Multimer. J. Bacteriol..

[13] Lamb J.R., Patel H., Montminy T., Wagner V.E., Iglewski B.H. (2003). FunctionalDomains of the RhlR Transcriptional Regulator ofPseudomonasaeruginosa. J. Bacteriol..

[14] Luo Z.-Q., Smyth A.J., Gao P., Qin Y., Farrand S.K. (2003). Mutational Analysis of TraR. J. Boil. Chem..

[15] Shadel G.S., Young R.F., Baldwin T.O. (1990). Use of regulated cell lysis in a lethal genetic selection in Escherichia coli: Identification of the autoinducer-binding region of the LuxR protein from Vibrio fischeri ATCC 7744. J. Bacteriol..

[16] Slock J., VanRiet D., Kolibachuk D., Greenberg E.P. (1990). Critical regions of the Vibrio fischeri luxR protein defined by mutational analysis. J. Bacteriol..

[17] Zhang R.-G., Pappas K.M., Brace J.L., Miller P.C., Oulmassov T., Molyneaux J.M., Anderson J.C., Bashkin J.K., Winans S.C., Joachimiak A. (2002). Structure of a bacterial quorum-sensing transcription factor complexed with pheromone and DNA. Nature.

[18] Müh U., Hare B.J., Duerkop B.A., Schuster M., Hanzelka B.L., Heim R., Olson E.R., Greenberg E.P. (2006). A structurally unrelated mimic of a Pseudomonas aeruginosa acyl-homoserine lactone quorum-sensing signal. Proc. Natl. Acad. Sci. USA.

[19] Koch B., Liljefors T., Persson T., Nielsen J., Kjelleberg S., Givskov M. (2005). The LuxR receptor: The sites of interaction with quorum-sensing signals and inhibitors. Microbiology.

[20] Ding X., Yin B., Qian L., Zeng Z., Yang Z., Li H., Lu Y., Zhou S. (2011). Screening for novel quorum-sensing inhibitors to interfere with the formation of Pseudomonas aeruginosa biofilm. J. Med. Microbiol..

[21] Grandclément C., Tannières M., Moréra S., Dessaux Y., Faure D. (2015). Quorum quenching: Role in nature and applied developments. FEMS Microbiol. Rev..

[22] Martin C.A., Hoven A.D., Cook A.M. (2008). Therapeutic frontiers: Preventing and treating infectious diseases by inhibiting bacterial quorum sensing. Eur. J. Clin. Microbiol. Infect. Dis..

[23] Kociolek M. (2009). Quorum-Sensing Inhibitors and Biofilms. Anti Infect. Agents Med. Chem..

[24] Choudhary S., Schmidt-Dannert C. (2010). Applications of quorum sensing in biotechnology. Appl. Microbiol. Biotechnol..

[25] Clatworthy A.E., Pierson E., Hung D.T. (2007). Targeting virulence: A new paradigm for antimicrobial therapy. Nat. Methods.

[26] Kalia V.C. (2013). Quorum sensing inhibitors: An overview. Biotechnol. Adv..

[27] Chen F., Gao Y., Chen X., Yu Z., Li X. (2013). Quorum Quenching Enzymes and Their Application in Degrading Signal Molecules to Block Quorum Sensing-Dependent Infection. Int. J. Mol. Sci..

[28] Wang Z., Yu P., Zhang G., Xu L., Wang D., Wang L., Zeng X., Wang Y. (2010). Design, synthesis and antibacterial activity of novel andrographolide derivatives. Bioorg. Med. Chem..

[29] Qin X., Thota G.K., Singh R., Balamurugan R., Goycoolea F.M. (2020). Synthetic homoserine lactone analogues as antagonists of bacterial quorum sensing. Bioorg. Chem..

[30] Yang L., Rybtke M.T., Jakobsen T.H., Hentzer M., Bjarnsholt T., Givskov M., Tolker-Nielsen T. (2009). Computer-Aided Identification of Recognised Drugs as Pseudomonas aeruginosa Quorum-Sensing Inhibitors. Antimicrob. Agents Chemother..

[31] Zeng Z., Qian L., Cao L., Tan H., Huang Y., Xue X., Shen Y., Zhou S. (2008). Virtual screening for novel quorum sensing inhibitors to eradicate biofilm formation of Pseudomonas aeruginosa. Appl. Microbiol. Biotechnol..

[32] Annapoorani A., Umamageswaran V., Parameswari R., Pandian S.K., Ravi A.V. (2012). Computational discovery of putative quorum sensing inhibitors against LasR and RhlR receptor proteins of Pseudomonas aeruginosa. J. Comput. Mol. Des..

[33] Tan S.Y.-Y., Chua S.L., Chen Y., Rice S.A., Kjelleberg S., Nielsen T.E., Yang L., Givskov M. (2013). Identification of Five Structurally Unrelated Quorum-Sensing Inhibitors of Pseudomonas aeruginosa from a Natural-Derivative Database. Antimicrob. Agents Chemother..

[34] Goodford P.J. (1985). A Computational procedure for determining energetically favorable binding sites on biologically important macromolecules. J. Med. Chem..

[35] Carosati E., Sciabola S., Cruciani G. (2004). Hydrogen Bonding Interactions of Covalently Bonded Fluorine Atoms: From Crystallographic Data to a New Angular Function in the GRID Force Field. J. Med. Chem..

[36] Ahlström M.M., Ridderström M., Luthman A.K., Zamora I. (2005). Virtual Screening and Scaffold Hopping Based on GRID Molecular Interaction Fields. J. Chem. Inf. Model..

[37] Sciabola S., Stanton R.V., Mills J.E., Flocco M.M., Baroni M., Cruciani G., Perruccio F., Mason J.S. (2009). High-Throughput Virtual Screening of Proteins Using GRID Molecular Interaction Fields. J. Chem. Inf. Model..

[38] Sanjurjo C.V., Engwer C., Qin X., Hembach L., Verdía-Cotelo T., Remuñán-López C., Vila-Sanjurjo A., Goycoolea F.M. (2016). A single intracellular protein governs the critical transition from an individual to a coordinated population response during quorum sensing: Origins of primordial language. bioRxiv.

[39] Skandamis P.N., Nychas G.-J. (2012). Quorum Sensing in the Context of Food Microbiology. Appl. Environ. Microbiol..

[40] Galloway W.R., Hodgkinson J.T., Bowden S., Welch M., Spring D.R. (2012). Applications of small molecule activators and inhibitors of quorum sensing in Gram-negative bacteria. Trends Microbiol..

[41] Takano E. (2006). γ-Butyrolactones: Streptomyces signalling molecules regulating antibiotic production and differentiation. Curr. Opin. Microbiol..

[42] Gram L., De Nys R., Maximilien R., Givskov M., Steinberg P., Kjelleberg S. (1996). Inhibitory Effects of Secondary Metabolites from the Red Alga Delisea pulchra on Swarming Motility of Proteus mirabilis. Appl. Environ. Microbiol..

[43] Givskov M., De Nys R., Manefield M., Gram L., Maximilien R., Eberl L., Molin S., Steinberg P., Kjelleberg S. (1996). Eukaryotic interference with homoserine lactone-mediated prokaryotuc signalling. J. Bacteriol..

[44] Manefield M., De Nys R., Naresh K., Roger R., Givskov M., Peter S., Kjelleberg S. (1999). Evidence that halogenated furanones from Delisea pulchra inhibit acylated homoserine lactone (AHL)-mediated gene expression by displacing the AHL signal from its receptor protein. Microbiology.

[45] Ren D., Sims J.J., Wood T.K. (2001). Inhibition of biofilm formation and swarming of Escherichia coli by (5Z)-4-bromo-5-(bromomethylene)-3-butyl-2(5H)-furanone. Environ. Microbiol..

[46] Defoirdt T., Miyamoto C.M., Wood T.K., Meighen E.A., Sorgeloos P., Verstraete W., Bossier P. (2007). The natural furanone (5Z)-4-bromo-5-(bromomethylene)-3-butyl-2(5H)-furanone disrupts quorum sensing-regulated gene expression in Vibrio harveyi by decreasing the DNA-binding activity of the transcriptional regulator protein luxR. Environ. Microbiol..

[47] Lönn-Stensrud J., Landin M.A., Benneche T., Petersen F.C., Scheie A.A. (2009). Furanones, potential agents for preventing Staphylococcus epidermidis biofilm infections?. J. Antimicrob. Chemother..

[48] Hentzer M., Wu H., Andersen J.B., Riedel K., Rasmussen T.B., Bagge N., Kumar N., Schembri M.A., Song Z., Kristoffersen P. (2003). Attenuation of Pseudomonas aeruginosa virulence by quorum sensing inhibitors. EMBO J..

[49] Manefield M., Rasmussen T.B., Henzter M., Andersen J.B., Steinberg P., Kjelleberg S., Givskov M. (2002). Halogenated furanones inhibit quorum sensing through accelerated LuxR turnover. Microbiology.

[50] Steenackers H.P., Levin J., Janssens J.C., De Weerdt A., Balzarini J., Vanderleyden J., De Vos D.E., De Keersmaecker S.C.J. (2010). Structure–activity relationship of brominated 3-alkyl-5-methylene-2(5H)-furanones and alkylmaleic anhydrides as inhibitors of Salmonella biofilm formation and quorum sensing regulated bioluminescence in Vibrio harveyi. Bioorganic Med. Chem..

[51] Janssens J.C.A., Steenackers H., Robijns S., Gellens E., Levin J., Zhao H., Hermans K., De Coster D., Verhoeven T.L., Marchal K. (2008). Brominated Furanones Inhibit Biofilm Formation by Salmonella enterica Serovar Typhimurium. Appl. Environ. Microbiol..

[52] Ponnusamy K., Paul D., Kweon J.H. (2009). Inhibition of Quorum Sensing Mechanism andAeromonas hydrophilaBiofilm Formation by Vanillin. Environ. Eng. Sci..

[53] Niu C., Afre S., Gilbert E.S. (2006). Subinhibitory concentrations of cinnamaldehyde interfere with quorum sensing. Lett. Appl. Microbiol..

[54] Brackman G., Defoirdt T., Miyamoto C., Bossier P., Van Calenbergh S., Nelis H.J., Coenye T. (2008). Cinnamaldehyde and cinnamaldehyde derivatives reduce virulence in Vibrio spp. by decreasing the DNA-binding activity of the quorum sensing response regulator LuxR. BMC Microbiol..

[55] Chang C.-Y., Krishnan T., Wang H., Chen Y., Yin W.-F., Chong Y.-M., Tan L.Y., Chong T.M., Chan K.-G. (2014). Non-antibiotic quorum sensing inhibitors acting against N-acyl homoserine lactone synthase as druggable target. Sci. Rep..

[56] Furuya T., Arai Y., Kino K. (2012). Biotechnological Production of Caffeic Acid by Bacterial Cytochrome P450 CYP199A2. Appl. Environ. Microbiol..

[57] Hayman M., Kam P.C. (2008). Capsaicin: A review of its pharmacology and clinical applications. Curr. Anaesth. Crit. Care.

[58] Jones N.L., Shabib S., Sherman P.M. (1997). Capsaicin as an inhibitor of the growth of the gastric pathogen Helicobacter pylori. FEMS Microbiol. Lett..

[59] Zeyrek F.Y., Oguz E. (2005). In vitro activity of capsaicin against Helicobacter pylori. Ann. Microbiol..

[60] Lee H.S., Lee S.Y., Park S.H., Lee J.H., Ahn S.K., Choi Y.M., Choi D.J., Chang J.-H. (2013). Antimicrobial medical sutures with caffeic acid phenethyl ester and their in vitro/in vivo biological assessment. MedChemComm.

[61] Murtaza G., Karim S., Akram M.R., Khan S.A., Azhar S., Mumtaz A., Bin Asad M.H.H. (2014). Caffeic Acid Phenethyl Ester and Therapeutic Potentials. BioMed Res. Int..

[62] Norizan S.N.M., Yin W.-F., Chan K.-G. (2013). Caffeine as a Potential Quorum Sensing Inhibitor. Sensors.

[63] Koo H.-J., Song Y.S., Kim H.-J., Lee Y.-H., Hong S.-M., Lim S.-J., Kim B.-C., Jin C., Lim C.-J., Park E.-H. (2004). Anti-inflammatory effects of genipin, an active principle of gardenia. Eur. J. Pharmacol..

[64] Kim B.-C., Kim H.-G., Lee S.-A., Lim S., Park E.-H., Kim S.-J., Lim C.-J. (2005). Genipin-induced apoptosis in hepatoma cells is mediated by reactive oxygen species/c-Jun NH2-terminal kinase-dependent activation of mitochondrial pathway. Biochem. Pharmacol..

[65] Sanjurjo C.V., David L., Remuñán-López C., Goycoolea F.M., Vila-Sanjurjo A. (2019). Effect of the ultrastructure of chitosan nanoparticles in colloidal stability, quorum quenching and antibacterial activities. J. Colloid Interface Sci..

[66] Lin Y.-H., Tsai S.-C., Lai C.-H., Lee C.-H., He Z.S., Tseng G.-C. (2013). Genipin-cross-linked fucose–chitosan/heparin nanoparticles for the eradication of Helicobacter pylori. Biomaterials.

[67] Hwang Y.-J., Larsen J., Krasieva T.B., Lyubovitsky J.G. (2011). Effect of Genipin Crosslinking on the Optical Spectral Properties and Structures of Collagen Hydrogels. ACS Appl. Mater. Interfaces.

[68] Canton B., Labno A., Endy D. (2008). Refinement and standardisation of synthetic biological parts and devices. Nat. Biotechnol..

[69] Pe’delacq J.-D., Cabantous S., Tran T., Terwilliger T.C., Waldo G.S. (2006). Corrigendum: Engineering and characterisation of a superfolder green fluorescent protein. Nat. Biotechnol..

[70] Lin J.-S., Cheng J., Wang Y., Shen X. (2018). The Pseudomonas Quinolone Signal (PQS): Not Just for Quorum Sensing Anymore. Front. Microbiol..

[71] Price-Whelan A., Dietrich L.E., Newman D.K. (2006). Rethinking ’secondary’ metabolism: Physiological roles for phenazine antibiotics. Nat. Methods.

[72] Pesci E.C., Milbank J.B.J., Pearson J.P., McKnight S., Kende A.S., Greenberg E.P., Iglewski B.H. (1999). Quinolone signaling in the cell-to-cell communication system of Pseudomonas aeruginosa. Proc. Natl. Acad. Sci. USA.

[73] McKnight S.L., Iglewski B.H., Pesci E.C. (2000). The Pseudomonas Quinolone Signal Regulates rhl Quorum Sensing in Pseudomonas aeruginosa. J. Bacteriol..

[74] Mukherjee S., Moustafa D.A., Stergioula V., Smith C.D., Goldberg J.B., Bassler B. (2018). The PqsE and RhlR proteins are an autoinducer synthase–receptor pair that control virulence and biofilm development in Pseudomonas aeruginosa. Proc. Natl. Acad. Sci. USA.

[75] Mahajan-Miklos S., Tan M., Rahme L.G., Ausubel F.M. (1999). Molecular Mechanisms of Bacterial Virulence Elucidated Using a Pseudomonas aeruginosa– Caenorhabditis elegans Pathogenesis Model. Cell.

[76] Dietrich L.E., Price-Whelan A., Petersen A., Whiteley M., Newman D.K. (2006). The phenazine pyocyanin is a terminal signalling factor in the quorum sensing network of Pseudomonas aeruginosa. Mol. Microbiol..

[77] Seo S., Gao Y., Kim N., Szubin R., Yang J., Cho B.-K., Palsson B.O. (2017). Revealing genome-scale transcriptional regulatory landscape of OmpR highlights its expanded regulatory roles under osmotic stress in Escherichia coli K-12 MG1655. Sci. Rep..

[78] Jagmann N., Brachvogel H.-P., Philipp B. (2010). Parastic growth of Pseudomonas aeruginosa in co-culture with the chitinolytic bacterium Aeromonas hydrophila. Environ. Microbiol..

[79] Bou-Abdallah F., Chasteen N.D., Lesser M.P. (2006). Quenching of superoxide radicals by green fluorecent protein. Biochim. Biophys. Acta.

[80] Mavrodi D.V., Bonsall R.F., Delaney S.M., Soule M.J., Phillips G., Thomashow L.S. (2001). Functional Analysis of Genes for Biosynthesis of Pyocyanin and Phenazine-1-Carboxamide from Pseudomonas aeruginosa PAO1. J. Bacteriol..

[81] Haynes W.C., Stodola F.H., Locke J.M., Pridham T.G., Conway H.F., Sohns V.E., Jackson R.W. (1956). PSEUDOMONAS AUREOFACIENS KLUYVER AND PHENAZINE α-CARBOXYLIC ACID, ITS CHARACTERISTIC PIGMENT. J. Bacteriol..

[82] Mazzola M., Cook R.J., Thomashow L.S., Weller D.M., Pierson L.S. (1992). Contribution of phenazine antibiotic biosynthesis to the ecological competence of fluorescent pseudomonads in soil habitats. Appl. Environ. Microbiol..

[83] Wang Y., Wilks J.C., Danhorn T., Ramos I., Croal L., Newman D.K. (2011). Phenazine-1-Carboxylic Acid Promotes Bacterial Biofilm Development via Ferrous Iron Acquisition. J. Bacteriol..

[84] Pierson L.S., Pierson E.A. (2010). Metabolism and function of phenazines in bacteria: Impacts on the behavior of bacteria in the environment and biotechnological processes. Appl. Microbiol. Biotechnol..

[85] Hassett D.J., Ma J.-F., Elkins J.G., McDermott T.R., Ochsner U.A., West S.E.H., Huang C.-T., Fredericks J., Burnett S., Stewart P.S. (1999). Quorum sensing in Pseudomonas aeruginosa controls expression of catalase and superoxide dismutase genes and mediates biofilm susceptibility to hydrogen peroxide. Mol. Microbiol..

[86] Morales D.K., Grahl N., Okegbe C., Dietrich L.E., Jacobs N.J., Hogan D.A. (2013). Control of Candida albicans Metabolism and Biofilm Formation by Pseudomonas aeruginosa Phenazines. mBio.

[87] Skindersoe M.E., Alhede M., Phipps R., Yang L., Jensen P.Ø., Rasmussen T.B., Bjarnsholt T., Tolker-Nielsen T., Høiby N., Givskov M. (2008). Effects of Antibiotics on Quorum Sensing in Pseudomonas aeruginosa. Antimicrob. Agents Chemother..

[88] Schertzer J.W., Boulette M.L., Whiteley M. (2009). More than a signal: Non-signaling properties of quorum sensing molecules. Trends Microbiol..

[89] Kubo I., Fujita K.-I., Lee S.H., Ha T.J. (2005). Antibacterial activity of polygodial. Phytother. Res..

[90] Wang X., Yao X., Zhu Z.-A., Tang T., Dai K., Sadovskaya I., Flahaut S., Jabbouri S. (2009). Effect of berberine on Staphylococcus epidermidis biofilm formation. Int. J. Antimicrob. Agents.

[91] Sun T., Li X.-D., Hong J., Liu C., Zhang X.-L., Zheng J.-P., Xu Y.-J., Ou Z.-Y., Zheng J.-L., Yu D.-J. (2019). Inhibitory Effect of Two Traditional Chinese Medicine Monomers, Berberine and Matrine, on the Quorum Sensing System of Antimicrobial-Resistant Escherichia coli. Front. Microbiol..

[92] Aswathanarayan J.B., Vittal R.R. (2018). Inhibition of biofilm formation and quorum sensing mediated phenotypes by berberine in Pseudomonas aeruginosa and Salmonella typhimurium. RSC Adv..

[93] Eiden F., Wendt R., Fenner H. (1978). ChemInform Abstract: PYRONES AND PYRIDONES, PART 74. QUINOLYLIDENE DERIVATIVES. Chem. Informationsdienst.

[94] Evans D., Eastwood F. (1974). Synthesis of an arylhydroxytetronimide and of 3-Hydroxy-4(1H)-quinolone derivatives. Aust. J. Chem..

[95] Cornforth J.W., James A.T. (1956). Structure of a naturally occurring antagonist of dihydrostreptomycin. Biochem. J..

