# Supplementary material for: Screening of Bacterial Quorum Sensing Inhibitors in a Vibrio fischeri LuxR-Based Synthetic Fluorescent E. coli Biosensor
Source: Pharmaceuticals (Basel). 2020 Sep 22;13(9):263. doi: 10.3390/ph13090263 (PMC7559085; doi:10.3390/ph13090263)
Supplement: Supplementary file 1 [file pharmaceuticals-13-00263-s001.pdf]

Supporting Information

Screening of Bacterial Quorum Sensing Inhibitors in a *Vibrio fischeri* LuxR-Based Synthetic Fluorescent *E. coli* Biosensor

Xiaofei Qin [1,2], Celina Vila-Sanjurjo [2,3], Ratna Singh [4], Bodo Philipp [5] and Francisco M. Goycoolea [2,6,*]

1 Department of Bioengineering, Zhuhai Campus of Zunyi Medical University, Zhuhai 519041, China; iamxfqin@njtech.edu.cn
2 Institute of Plant Biology and Biotechnology, Laboratory of Nanobiotechnology, University of Münster, Schlossplatz 8, D-48143 Münster, Germany
3 Department of Pharmacology, Pharmacy and Pharmaceutical Technology, University of Santiago de Compostela. Campus Vida, s/n, 15782 Santiago de Compostela, Spain; celina.vila@usc.es
4 Institute of Plant Biology and Biotechnology, Laboratory of Molecular Phytopathology and Renewable Resources, University of Münster, Schlossplatz 8, D-48143 Münster, Germany; singhr@uni-muenster.de
5 Institute of Molecular Microbiology and Biotechnology, University of Münster, Corrensstraße 3, D-48149 Münster, Germany; bodo.philipp@uni-muenster.de
6 School of Food Science and Nutrition, University of Leeds, Leeds LS2 9JT, United Kingdom
* Correspondence: F.M.Goycoolea@leeds.ac.uk; Tel.: +44-1133-431412

Additional experiments on response inhibition by pyocyanin and genipin using the fluorescence *E. coli* Top10 pSB1A3-T9002 biosensor strain and the control- constitutive GFP expressing *E. coli* Top10 pBCA9445-jtk28282::sfGFP strain:

The *E. coli* strain Top10 (Invitrogen, Life Technologies Co., UK) was transformed with plasmid pBCA9445-jtk2828, carrying a superfolder version of the *gfp* gene (*sfgfp*) [1], which was kindly donated by Prof. Anderson Lab (UC Berkeley, USA). The transformed strain expresses sfGFP constitutively and was used as control culture to test possible fluorescence quenching artifacts of genipin (GNP) and pyocyanin (PYO) that could account for the effects observed in the fluorescence *E. coli* Top10 pSB1A3-BBaT9002 biosensor (main text). Glycerol stocks from single-colony cultures of the *E. coli* Top10 pBCA9445-jtk28282::sfGFP and the *E. coli* Top10 pSB1A3-BBaT9002 biosensor strain were prepared as explained in the main text (Materials and Methods). Prior to each experiment, a glycerol stock from the single colony-culture of *E. coli* Top10 pBCA9445-jtk28282::sfGFP was diluted by a factor of 10^{-3} into 20 mL of supplemented M9 minimal medium and ampicillin (200 μg/mL) and incubated overnight at 37 °C under shaking (100 rpm). The overnight culture was then sub-diluted by a factor of $1x10^{-3}$ or $2x10^{-3}$ in 20 mL of supplemented M9 minimal medium and grown to an OD600 of ~0.0 (~4 h). Glycerol stocks of *E. coli* Top10 pSB1A3-BBaT9002 were diluted in supplemented M9 minimal medium prior each experiment as explained in the main text (Materials and Methods). A comparison of the fluorescence profiles of *E. coli* Top10 pSB1A3-BBaT9002 and *E. coli* Top10 pBCA9445-jtk28282::sfGFP is depicted in Figure S1.
Ten-μL aliquots of aqueous mixtures of PYO and GNP at specific concentrations were inoculated in the wells of a 96-well microplate containing 190 μL of the *E. coli* Top10 pBCA9445-jtk2828::sfGFP cultures or *E. coli* Top10 pSB1A3-BBaT9002 cultures, the latter in the presence of $5x10^{-10}$ M or $2.5x10^{-10}$M AHL, grown in supplemented M9 minimal medium as explained above. The microplate was incubated in the microplate reader and FI and OD_{600} were recorded over time as explained in the main text (Materials and Methods). The results of these experiments are depicted in Figures S2-S6.

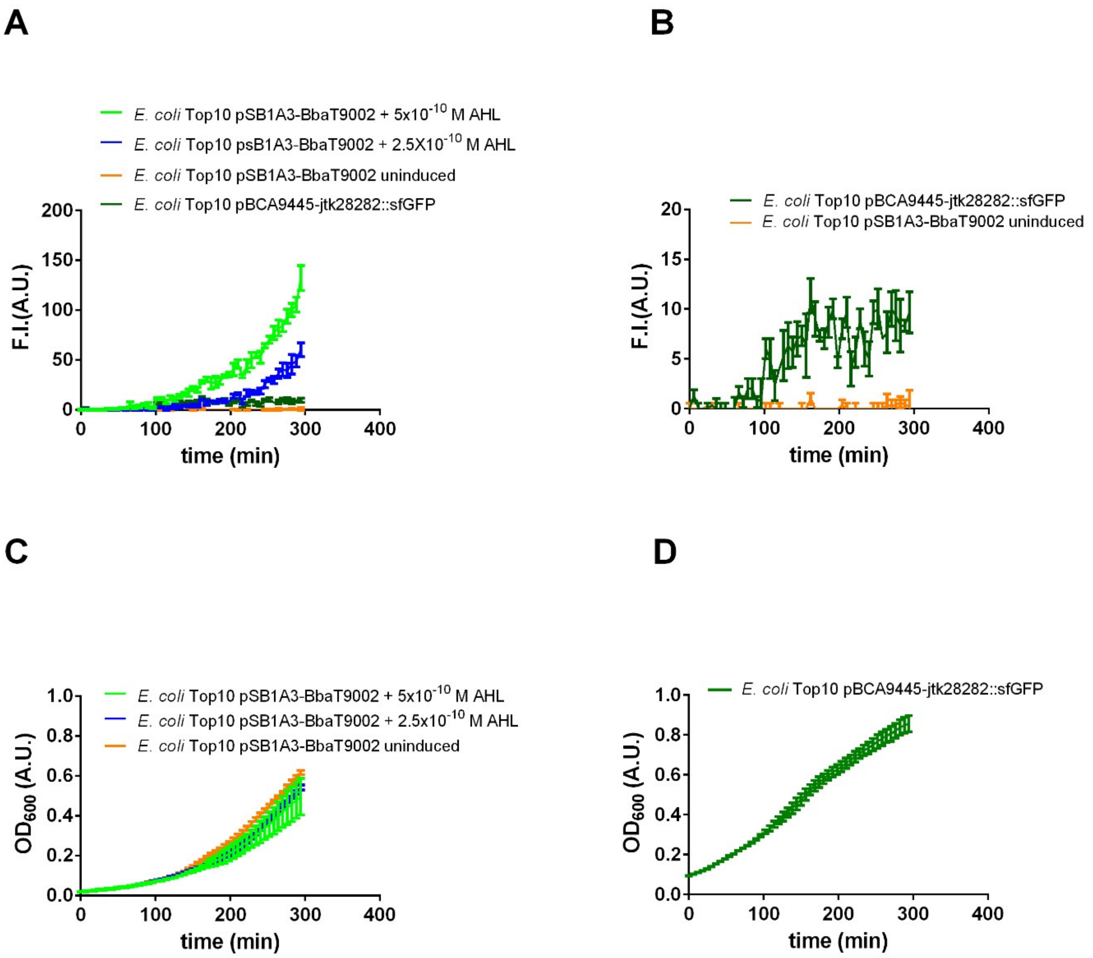

Figure S1. Comparison of the fluorescence response (**A, B**) and growth (**C, D**) of AHL-induced and uninduced *E. coli* Top10 pSB1A3-T9002 biosensor and *E. coli* Top10 pBCA9445-jtk2828::sfGFP. Data represent the mean and standard deviation of a single experiment with three biological replicates.

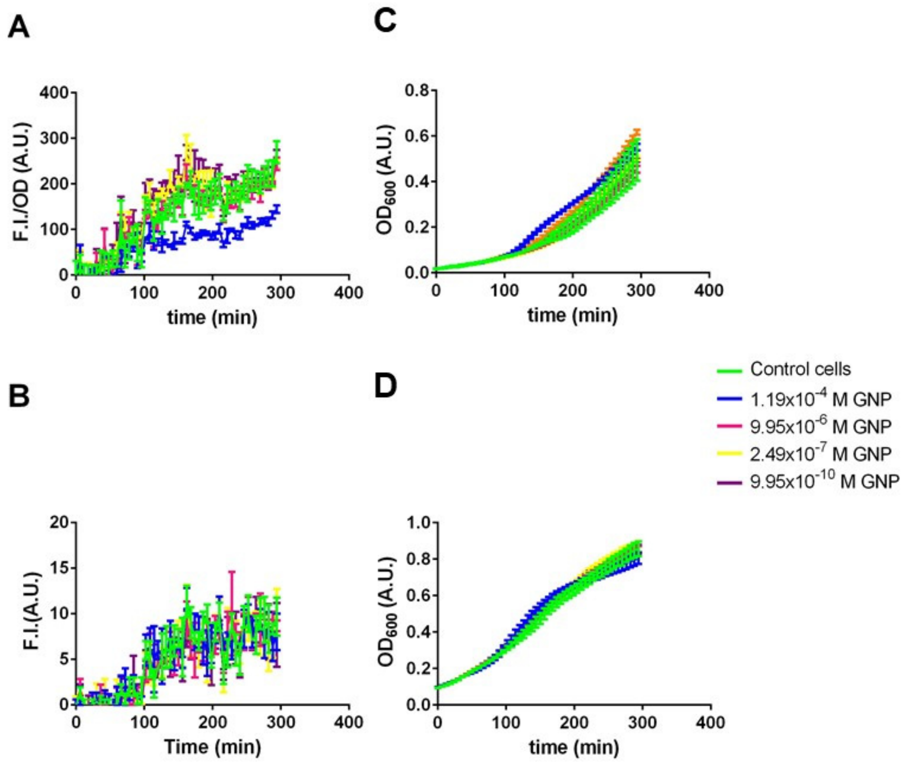

Figure S2. Comparison of the fluorescence response of AHL-induced (5x10^{-10} M AHL) *E. coli* Top10 pSB1A3-T9002 biosensor (A) and *E. coli* Top10 pBCA9445-jtk2828::sfGFP cultures (B) in the presence of various concentrations of GNP. (C-D) Corresponding growth curves. Data represent the mean and standard deviation of a single experiment with three biological replicates.

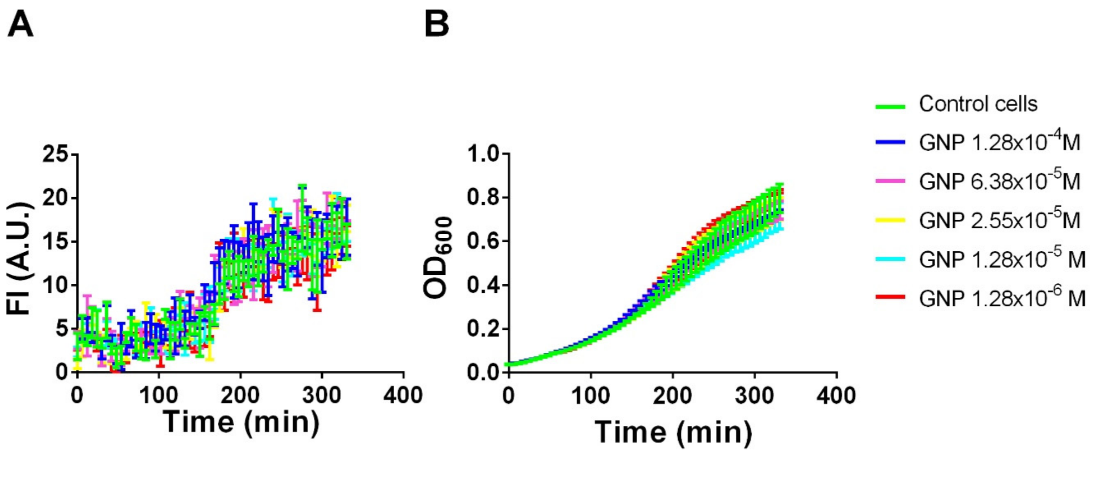

Figure S3. Effect of GNP at various concentrations on the constitutive fluorescence (A) and growth (B) of the *E. coli* Top10 pBCA9445-jtk28282::sfGFP strain. Data represent the mean and standard deviation of a single experiment with three biological replicates.

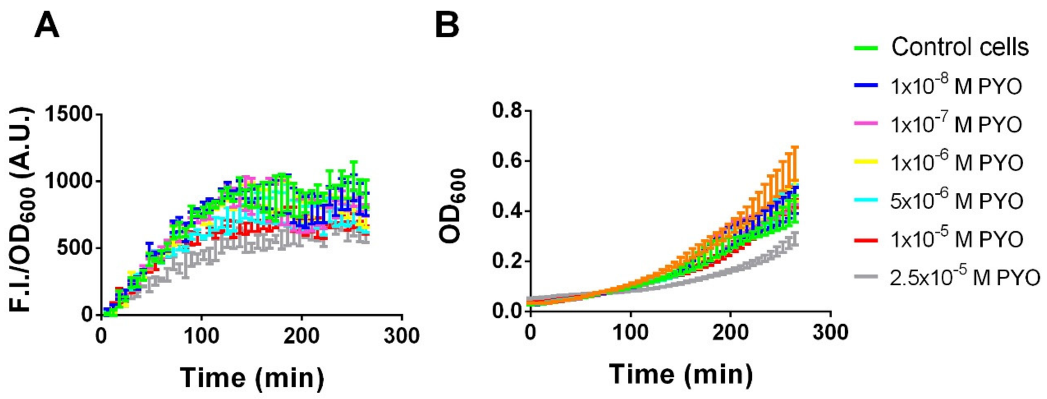

Figure S4. Effect of PYO at various concentrations on the density-normalized fluorescence of *E. coli* Top10 pSB1A3-T9002 biosensor cells in the presence of $2.5\text{x}10^{-10}$M AHL (A). B. Growth plots corresponding to the treatments in A. Data represent the mean and standard deviation of a single experiment with three biological replicates.

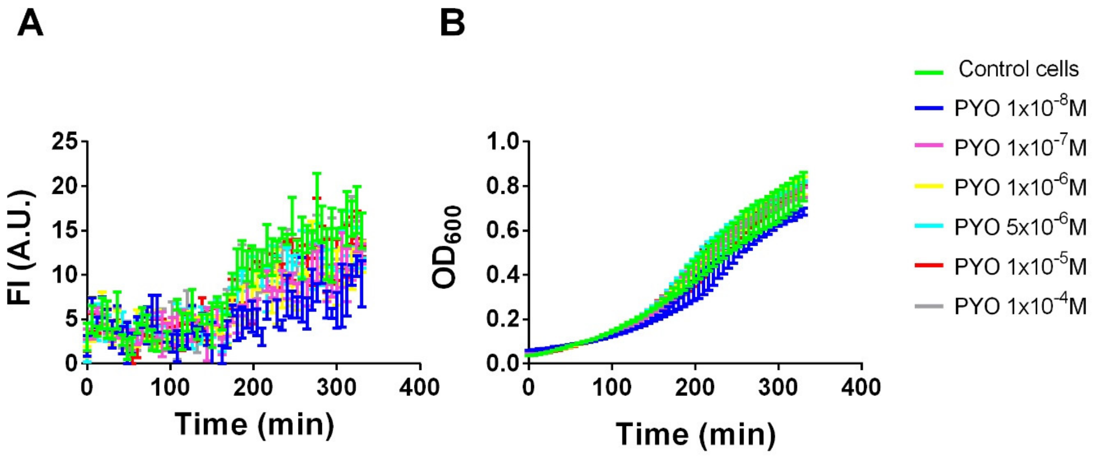

Figure S5. Effect of PYO at various concentrations on the constitutive fluorescence (A) and growth (B) of the *E. coli* Top10 pBCA9445-jtk28282::sfGFP strain. Data represent the mean and standard deviation of a single experiment with three biological replicates.

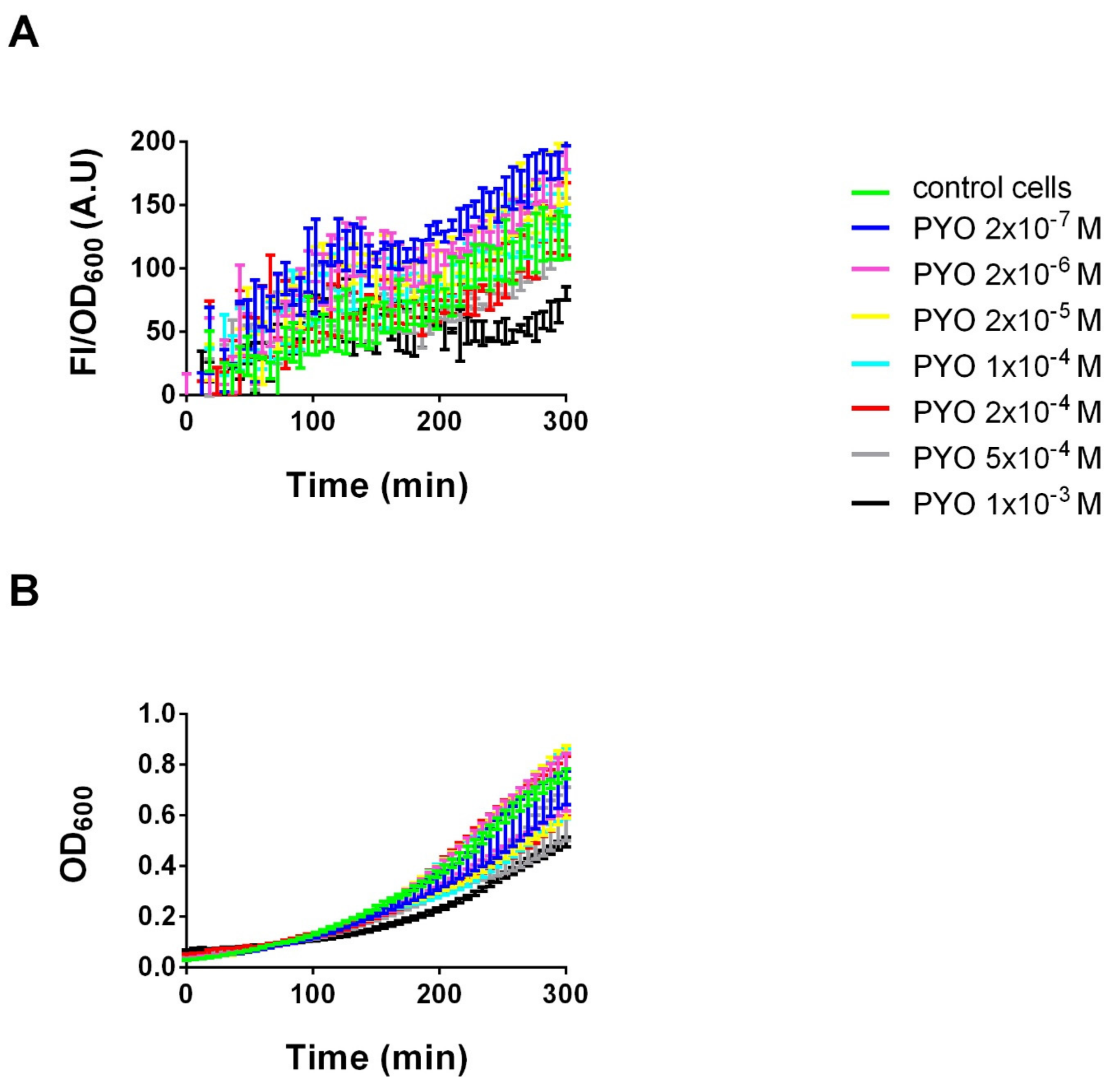

Figure S6. Effect of PYO at various concentrations on the density-normalized fluorescence of *E. coli* Top10 pSB1A3-T9002 biosensor cells in the presence of 2.5x10^{-10}M AHL (A). B. Corresponding growth plots. Data represent the mean and standard deviation of a single experiment with three biological replicates.

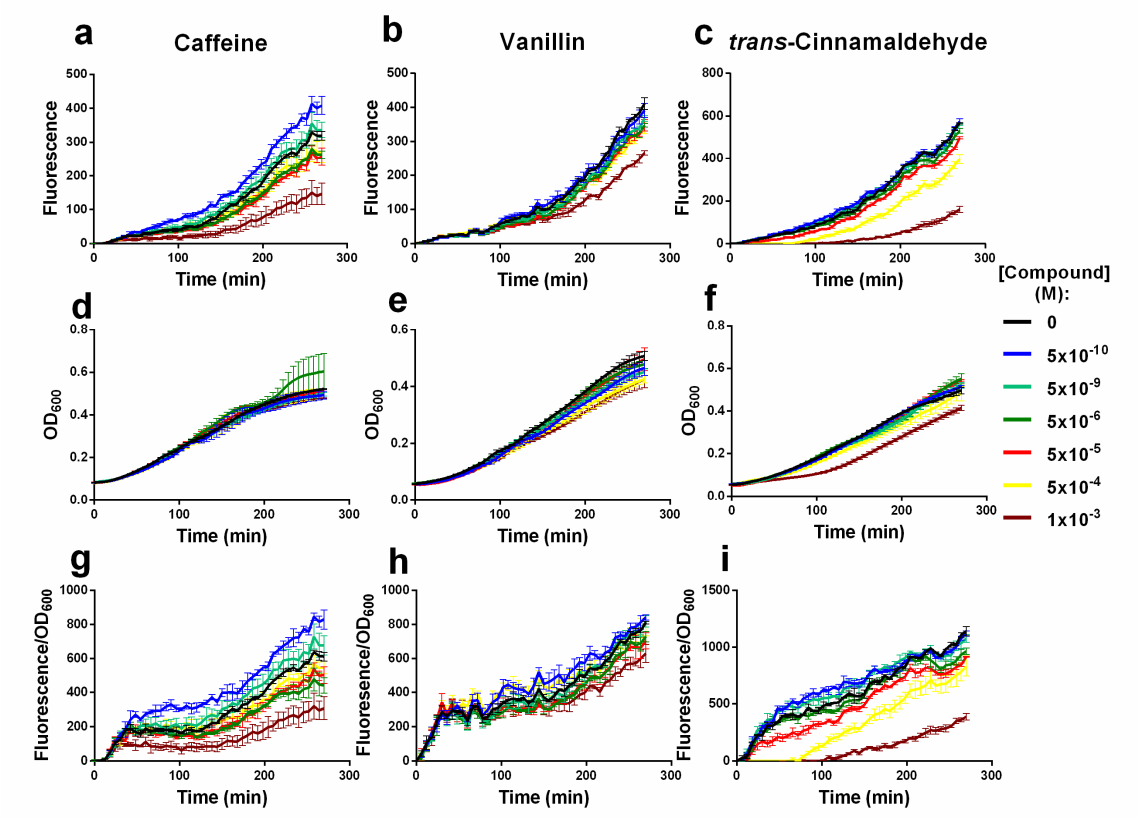

Figure S7. Effect of increasing concentrations (from $5x10^{-10}$ M to $1x10^{-3}$ M) of caffeine, vanillin and *trans*-cinnamaldehyde on the fluorescence (a-c), growth (d-f) and density-normalized fluorescence (g-i) of the *E. coli* biosensor over time. The three compounds are QS inhibiters in the absence of growth. The experiments were performed in triplicate.

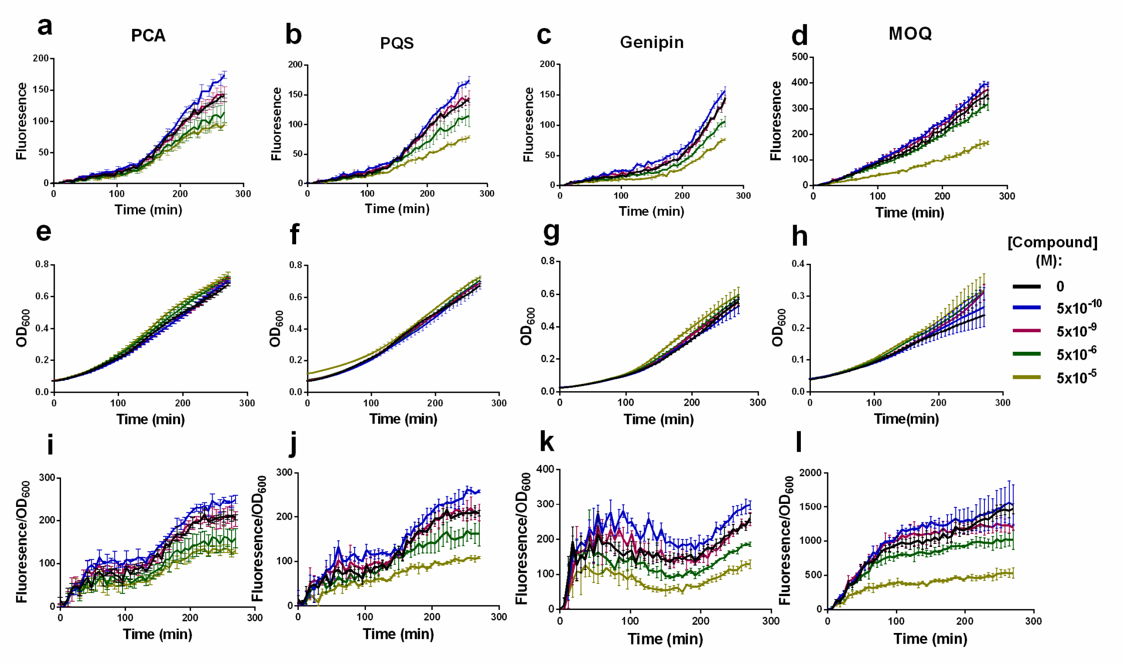

Figure S8. Effect of increasing concentrations (from 5x10^{-10} M to 5x10^{-5} M) of PCA, PQS, genipin and MOQ on the fluorescence (a-d), growth (e-h) and density-normalized fluorescence (i-l) of the *E. coli* biosensor over time. The four compounds are QS inhibiters in the absence of growth. The experiments were performed in triplicate.

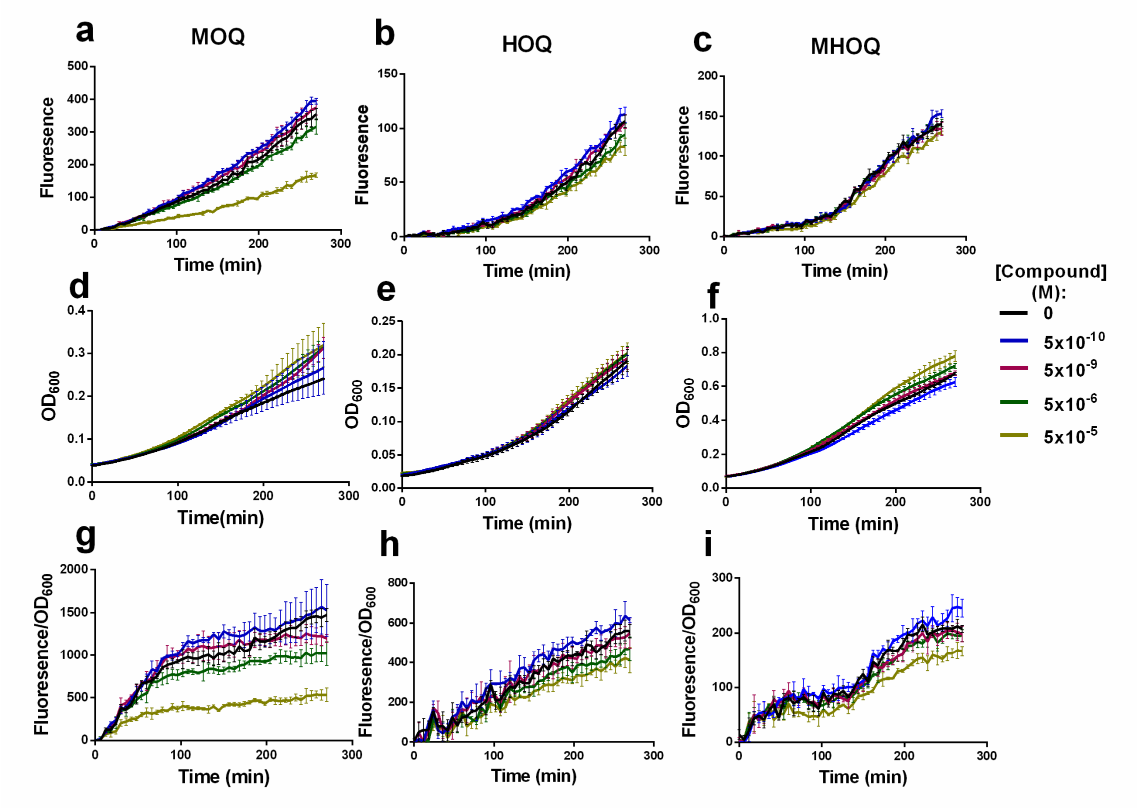

Figure S9. Effect of increasing concentrations (from 5x10^{-10} M to 5x10^{-5} M) of MOQ, HOQ and MHOQ on the fluorescence (a-c), growth (d-f) and density-normalized fluorescence (g-i) of the *E. coli* biosensor over time. Only compound MOQ is QS inhibiter in the absence of growth (MOQ; a, d, g). The experiments were performed in triplicate.

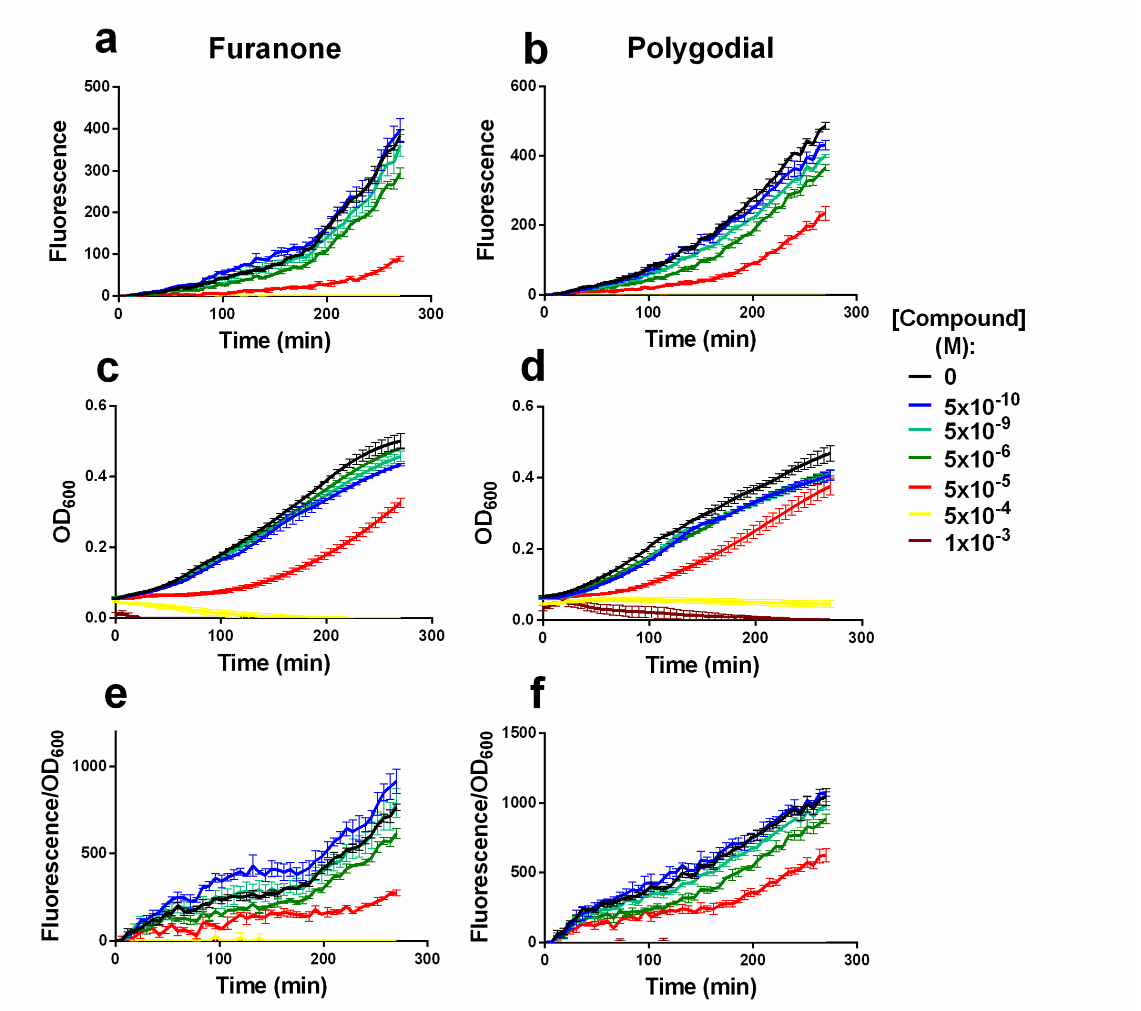

Figure S10. Effect of increasing concentrations (from 5x10^{-10} M to 1x10^{-3} M) of Furanone and polygodial on the fluorescence (a-b), growth (c-d) and density-normalized fluorescence (e-f) of the *E. coli* biosensor over time. The two compounds exhibited antibacterial activity the ability to reduce the QS response at an expense of hampering cell growth. The experiments were performed in triplicate.

References

1. Pedelacq, J-D.; Cabantous, S.; Tran, T.; Terwilliger, T. C.; Waldo, G. S. Engineering and characterization of a superfolder green fluorescent protein. Nat Biotechnol. 2006, 24, 79-88.
